# Evolutionary Relationships between *Rhynchosporium lolii* sp. nov. and Other *Rhynchosporium* Species on Grasses

**DOI:** 10.1371/journal.pone.0072536

**Published:** 2013-10-16

**Authors:** Kevin M. King, Jonathan S. West, Patrick C. Brunner, Paul S. Dyer, Bruce D. L. Fitt

**Affiliations:** 1 Department of Plant Biology and Crop Science, Rothamsted Research, Harpenden, Hertfordshire, United Kingdom; 2 School of Biology, University of Nottingham, Nottingham, United Kingdom; 3 School of Life and Medical Sciences, University of Hertfordshire, Hatfield, Hertfordshire, United Kingdom; 4 Plant Pathology Group, ETH Zurich, Zurich, Switzerland; University of Exeter Medical School, United Kingdom

## Abstract

The fungal genus *Rhynchosporium* (causative agent of leaf blotch) contains several host-specialised species, including *R. commune* (colonising barley and brome-grass), *R. agropyri* (couch-grass), *R. secalis* (rye and triticale) and the more distantly related *R. orthosporum* (cocksfoot). This study used molecular fingerprinting, multilocus DNA sequence data, conidial morphology, host range tests and scanning electron microscopy to investigate the relationship between *Rhynchosporium* species on ryegrasses, both economically important forage grasses and common wild grasses in many cereal growing areas, and other plant species. Two different types of *Rhynchosporium* were found on ryegrasses in the UK. Firstly, there were isolates of *R. commune* that were pathogenic to both barley and Italian ryegrass. Secondly, there were isolates of a new species, here named *R. lolii*, that were pathogenic only to ryegrass species. *R. lolii* was most closely related to *R. orthosporum*, but exhibited clear molecular, morphological and host range differences. The species was estimated to have diverged from *R. orthosporum* ca. 5735 years before the present. The colonisation strategy of all of the different *Rhynchosporium* species involved extensive hyphal growth in the sub-cuticular regions of the leaves. Finally, new species-specific PCR diagnostic tests were developed that could distinguish between these five closely related *Rhynchosporium* species.

## Introduction

Leaf blotch (scald) caused by the fungal pathogen *Rhynchosporium commune* is an economically important disease of barley (*Hordeum vulgare*) crops throughout the world [1] with yield losses of 1–10% common [2]. United Kingdom (UK) survey data indicates that barley crops grown in 2005 had 0.6% area with rhynchosporium lesions on leaf two at growth stage 75 (medium milk development stage; [3]). This equates to an estimated UK national yield loss of £10.8 million per annum (at a price of £225/tonne) despite fungicide treatment [4]. The symptoms of *Rhynchosporium* colonisation of barley can include coalescing lesions with dark brown margins and pale green or pale brown centres [5].

Phylogeographical analyses of *R. commune* isolates obtained from barley across five continents led to the conclusion that the pathogen did not emerge in association with its current barley host, believed to have been first domesticated in the ‘Fertile Crescent of the Middle East about 10,000 years before the present [6]. Instead, data from phylogenetic analyses using the *R. commune* avirulence gene *NIP1* and flanking regions [7] suggested that modern populations of *R. commune* originated in northern Europe approximately 2500–5000 years ago, when the pathogen switched from a wild grass species onto cultivated barley shortly after barley was introduced there, and that it subsequently spread into other barley-growing areas of the world.

The *Rhynchosporium* pathogen is also found on a number of other graminaceous hosts, including rye (*Secale cereale*), triticale (x *Triticosecale*), cocksfoot (*Dactylis glomerata*) and Italian and perennial ryegrasses (*Lolium multiflorum* and *Lolium perenne*, respectively) [8], [9], [10], [11]. Insights into the evolutionary history of the *Rhynchosporium* pathogen on different hosts were provided by Zaffarano *et al*. [12. 13], who used sequencing of several gene loci and host range testing experiments to demonstrate that the genus *Rhynchosporium* is comprised of at least four closely-related, but host-specialised, species. Three of the species produce conidia terminating with an oblique point, termed beak-shaped conidia [14]; (i) *R. commune* causing leaf blotch symptoms on barley, wall barley (*Hordeum murinum*), wild barley (*Hordeum spontaneum*), barley grass (*Hordeum glaucum*, *Hordeum leporinum*) and brome-grass (*Bromus diandrus*); (ii) *R. agropyri* on bearded couch-grass (*Agropyron caninum*) and couch-grass (*Agropyron repens*); (iii) *R. secalis* on rye and triticale. The remaining species *R. orthosporum*, which is more distantly related [13], produces cylindrically-shaped conidia [14] and is specialised on cocksfoot.

One limitation of the studies by Zaffarano *et al*. [12], [13] is that *Rhynchosporium* isolates from ryegrasses were not included. These grasses are both economically important forage grasses and commonly occurring weeds of cereal crops on most continents, and although originally native to Europe, Asia and North Africa they have now been introduced into almost all temperate countries of the world [11], [15], [16]. A previous investigation [11] had identified two types of *Rhynchosporium* that could cause leaf blotch on ryegrasses, with differences between them in conidial shape and host range. However, it is at present unclear how *Rhynchosporium* isolates from ryegrasses are related to the four described fungal species [13], none of which included ryegrass species in host range definitions Therefore, there is a need to characterise isolates from ryegrasses to determine their relatedness to the *Rhynchosporium* species of Zaffarano *et al*. [13]. Such characterisation is essential to understand any potential role for ryegrass species as a source of *Rhynchosporium* inoculum able to initiate leaf blotch epidemics on barley crops.

Currently, only very limited PCR (polymerase chain reaction)-based methods are available to distinguish between the four *Rhynchosporium* species described by Zaffarano *et al*. [13]. A restriction fragment length polymorphism diagnostic was developed [13] that could discriminate between isolates of *R. commune* and *R. secalis*. However, this test is time-consuming due to the requirement for an additional restriction digest step following PCR. Apart from this test, the species can be distinguished only at a molecular level on the basis of single nucleotide polymorphisms. The development of species-specific PCR diagnostic tests would provide a valuable tool to directly confirm species identity of isolates and to determine species distribution in field samples of different grass hosts.

Little is currently known about the how the four recently described *Rhynchosporium* species of Zaffarano *et al*. [13] might differ in the manner by which they colonise their plant hosts. For example, only *R. commune* colonisation of barley [17] and *R. orthosporum* colonisation of cocksfoot [10] have been the subject of detailed microscopical investigations. On both hosts, *Rhynchosporium* hyphae grew extracellularly (i.e. outside the plant cells) and extensive colonisation of the leaf sub-cuticular area was observed. Although Caldwell [14] made some observations about the colonisation strategy of *Rhynchosporium* on other grass species, no microscopic evidence is currently available to determine whether *R. agropyri*, *R. secalis* and the pathogen on ryegrasses all colonise the same sub-cuticular niche of their respective grass hosts.

This paper reports work to further investigate the different *Rhynchosporium* species on various grass hosts. Specifically, it uses a combination of molecular, morphological, pathological and microscopy approaches to (i) report the discovery of *R. commune* isolates that are pathogenic to both barley and Italian ryegrass; (ii) describe a new species that is specialised only to ryegrass species; (iii) report results of both microscopic and molecular investigations into the different host-specialised *Rhynchosporium* species.

## Methods

### Ethics Statement

Diseased plant material was collected from plots (permission obtained from IBERS-Aberystwyth University, NIAB-TAG and Rothamsted Research, all based in the UK). Diseased plant material and fungal cultures were imported into the UK under Defra plant health license number PHL174G/6192(10/2009).

### Isolation of the *Rhynchosporium* Pathogen

Leaves of barley, couch-grass, cocksfoot, Italian ryegrass and perennial ryegrass with distinct leaf blotch lesions were collected during the period of 2009–2011 from plots, samples being collected at least ∼1 metre apart within sites. To isolate *Rhynchosporium*, leaf blotch lesions were cut from green leaves, rinsed first in a 70% ethanol (v/v) solution for 2 min, followed by a 10% sodium hypochlorite (v/v) solution (minimum 8% available chlorine; Fisher Scientific, UK) for 5 min, and finished by a wash in sterile distilled water for 1 min. Lesions were then dried between pieces of sterile tissue paper and incubated at 18°C on Czapek dox plates (Sigma Aldrich, UK) amended to include 0.5% mycological peptone (Oxoid, UK) and penicillin and streptomycin sulphate at a final concentration of 100 and 50 parts per million, respectively. After 3 days, mycelium growing out from lesion margins was excised and used to establish single conidial cultures on lima bean agar (LBA; Difco, UK), which were grown at 18°C for 10 days.

### Fungal Isolates Obtained and Long-term Storage Procedure

A total of 44 *Rhynchosporium* isolates were obtained from leaves with leaf blotch lesions collected from five sites in the UK and two sites in Romania. New isolates collected included 22 isolates from Italian or perennial ryegrass in the UK, 11 isolates from couch-grass in Romania and the UK, seven isolates from cocksfoot in the UK and four isolates from barley in the UK. An additional 49 isolates, including representatives of *R. commune*, *R. agropyri*, *R. secalis* and *R. orthosporum*, obtained from international collaborators, had previously been isolated from various field sites most of which were throughout Europe. Collaborators included Dr Louise Cooke (Agri-Food and Biosciences Institute, UK), Dr James Fountaine (Scotland’s Rural College, UK), Dr Nichola Hawkins (Rothamsted Research, UK), Dr Wolfgang Knogge (Leibniz Institute of Plant Biochemistry, Germany) and Prof Bruce McDonald and Dr Tryggvi Stefansson (ETH Zürich, Switzerland). All isolates were then stored as silica stocks at −80°C [18].

### DNA Extraction

All fungal isolates were revived from −80°C storage by dispensing small amounts of silica stock onto potato dextrose agar (PDA, Oxoid, UK) plates overlaid with a single cellulose disk (A.A. Packaging Ltd, UK). Plates were sealed with a double layer of parafilm (Pechiney Plastic Packaging, USA) and incubated at 18°C in the dark for 10–15 days. After this period, fungal mycelium was scraped from the surface of the overlaid cellulose disk and DNA was extracted from lyophilized tissue using a DNeasy extraction kit (Qiagen, UK), according to the manufacturer’s instructions.

### RAPD-PCR and Rep-PCR Genomic Fingerprinting

Seventy-nine *Rhynchosporium* isolates were examined by random amplification of polymorphic DNA PCR (RAPD-PCR) fingerprinting (Table S1) as described in Murtagh *et al*. [19], except that a TC-512 programmable thermal controller (Techne, UK) was used for all PCR. Seven RAPD-PCR primers (Operon Technologies, UK; Table S2) were used; in preliminary testing they had been found to generate polymorphisms with a subset of *Rhynchosporium* isolates. No template controls were included for use with all RAPD-PCR sets and selected isolates from each of the different species were tested in duplicate to ensure that results were reproducible. PCR products (10 µl) were separated by gel electrophoresis on 1.5% agarose gels in 1×Tris-Borate-EDTA (TBE) buffer (National Diagnostics, UK) and stained with an ethidium bromide solution (200 µl of a 1 mg/ml ethidium bromide per 100 ml of 1×TBE buffer). Amplicons were viewed on a transilluminator and digital images obtained (Gene Genius Bio Imaging System, Syngene, Synoptics Ltd, UK). Unambiguous bands were chosen for scoring and their presence or absence was recorded in binary form (1 = present, 0 = absent), with data from all seven RAPD-PCR primers combined in the analyses. A neighbour joining analysis was made using Jaccard’s coefficient and a boot-strapped phylogram (based on 1,000 repeats of the tree) was produced using FreeTree software [20]. A text version of the dendrogram of the tree was exported to TreeView software [21].

Seventy-one *Rhynchosporium* isolates were examined with repetitive-sequence-based PCR (rep-PCR) genomic fingerprinting (Table S1) using primer pair combinations ERIC2/BOXA1R and ERICF/BOXA1R [22], [23] (Table S2). No template controls were included for use with both primer pairs and selected isolates from each of the different species were tested in duplicate to ensure that results were reproducible. Reactions were carried out in 20 µl volumes, each containing 10 µl Jumpstart RedTaq mastermix (Sigma Aldrich, UK), 7 µl sterile distilled water, 1 µl each of both primers (10 pmol µl^−1^ stock) and 1 µl of template DNA (10 ng). For testing of five isolates (RS04ITA D-2.2, RS04ITA D-3.1, RS04ITA D-4.1, RS04ITA D-6.1, RS04ITA D-6.2), 10 µl of template DNA (total 10 ng) was added (achieved by increasing overall reaction volumes) because only dilute concentrations of template DNA were available. PCR was carried out using a PTC-100 programmable thermal controller (MJ Research, USA) and reaction conditions were an initial hold at 96°C for 2 min, followed by 35 cycles of 94°C for 30 sec, 52°C for 1 min and 65°C for 5 min. PCR products were then analysed as described for RAPD-PCR products.

### Phylogenetic Analyses

Partial sequences of the alpha-tubulin, beta-tubulin and ITS (internal transcribed spacer) gene loci were obtained for ten isolates (2lm11, 3ar10, 6ar10 and 10ar10, 59dg09, Rs04ITA D-6.2, 4lm11, 7lm11, 13lp11 and 15lp11; isolate details and accompanying GenBank accession numbers provided in Table S3) using primers (Table S2) and reaction conditions described by Zaffarano *et al*. [12]. No template controls were included in reactions to amplify the three gene loci. Jumpstart high fidelity mix (Roche, Germany) was used in all reactions, with reaction components selected according to the manufacturer’s instructions with the following modifications; no DMSO was included and each reaction included 10 mM of each dNTP (Fermentas, UK) and 10–25 ng of template DNA. PCR products were visualised on a 1% agarose gel to ensure the presence of a single amplicon and purified using a MinElute kit (Qiagen, UK) according to the manufacturer’s instructions. DNA was sent to Eurofin MWG Operon for bi-directional sequencing using an ABI 3730XL machine, with the exception of the beta-tubulin PCR product for which only primer BTUB21F was used.

Individual sequences were imported into the BioEdit Sequence Alignment Editor (version 7.0.9.0; [24]) and trace sequence data of poor read quality were removed. Sequences were imported into the Geneious Pro 5.5.6 software package and the partial alpha-tubulin, beta-tubulin and ITS (partial 18S rRNA, ITS1, 5.8S rRNA, partial ITS2) sequences were edited to 1538, 542 and 492 bases in length (including gaps), respectively. For the ten isolates partially sequenced in the present study, sequence data of all three gene loci were concatenated. The CLUSTAL W algorithm contained in the Geneious software package was then used to align these sequences with the concatenated haplotype sequences of *R. commune*, *R. agropyri*, *R. secalis* and *R. orthosporum* obtained by Zaffarano *et al*. [12].

The relationship of isolates and haplotypes was inferred using the coalescent-based Bayesian Markov Chain Monte Carlo (MCMC) method implemented in the program BEAST version 1.4.1 [25]. To allow a direct comparison with previous studies, a strict molecular clock model was applied and the phylogenetic tree was internally calibrated by assuming a time-to-most-recent-common-ancestor (TMRCA) of 2487–4791 years (mean 3625) as inferred for the cluster *R commune/R. agropyri/R. secalis* [12]. The MCMC analysis was run for 10^7^ generations, sampling every 1000th iteration after an initial burn-in of 10%. The performance of the MCMC process was checked for stationarity and large effective sample sizes in TRACER (available from http://beast.bio.ed.ac.uk/Tracer). A maximum clade credibility tree was constructed after discarding the first 10% of inferred trees. The mean and corresponding credibility intervals of the estimated TMRCAs were depicted using TRACER.

### Microscopic Analysis of Conidial Morphology

Forty isolates, including isolates of *R. commune*, *R. agropyri*, *R. secalis*, *R. orthosporum* or *Rhynchosporium* isolates collected from ryegrasses (Table S1), were grown on LBA plates at 15°C in the dark. After 10 days of growth, sterile distilled water (2 ml) was added to each LBA plate and conidia were dislodged using an L-shaped sterile plastic spreader. Conidial suspensions were placed in sterile 2 ml microfuge tubes after filtration through two layers of sterile muslin to remove mycelial fragments. Conidial dimensions of 26 isolates were measured using fresh (within 8 hours of harvest) conidial suspensions maintained on ice (∼0°C), while conidial suspensions of 18 isolates (including four that were also measured fresh) were stored at −20°C and measured later. The shape of individual conidia of individual isolates was recorded as either beak-shaped or cylindrically-shaped [14]. Both the length and width of 25 mature (defined as having two cells clearly divided by a septum) conidia were measured with a digital CCD camera (Hamamatsu C8484 05G01) using *HC*image software (Hamamatsu Photonics K.K., Japan).

Light microscopic images of representative conidia of 17 isolates were also made on a Zeiss axiophot light microscope; images were obtained using a QImaging monochrome camera equipped with a Retiga XEi liquid crystal RGB filter and operated using MetaMorph ver. 7.6 software. Before statistical analysis of data, isolates were separated into two different groups; those with beak-shaped conidia (*R. commune* including two isolates from Italian ryegrass, *R. agropyri* and *R. secalis*) and those with cylindrically-shaped conidia (*R. orthosporum* and *Rhynchosporium* isolates from ryegrasses). Data were analysed using ANOVA (GenStat version 14; [26]).

### Host Range Testing

Twenty-two *Rhynchosporium* isolates (Table 1) were revived from −80°C silica stocks onto LBA plates as described previously in the text, with plates sealed with a double layer of parafilm and grown at 18°C in the dark for 10–12 days. Conidia were harvested from LBA plates as described previously in the text, and concentrations of suspensions were determined using a haemocytometer (Improved Neubauer haemocytometer, Weber Scientific International, UK). Aliquots (5–10 ml) of fungal inoculum (5×10^5^ conidia ml^−1^) were prepared using sterile distilled water (containing 0.01% Tween 80; Fisher Scientific, UK) in 50 ml Falcon tubes (CellStar, USA) and frozen at −20°C until required.

**Table 1:** Host range experiments suggest general host-specialisation of *Rhynchosporium* species.

					Development of leaf blotch lesionsa, b			
Species	Isolate	Original host	Origin	Collected	Barley^c^	Cocksfoot^d^	Italian ryegrass^e^	Perennial ryegrass^e^
*R. commune*	19hv09	Barley	Hertfordshire, UK	2009	+ +	− −	− −, − −	− −, − −
*R. commune*	53hv09	Barley	Hertfordshire, UK	2009	+ +	− −	− −, − −	− −, − −
*R. commune*	73hv09	Barley	Hertfordshire, UK	2009	+ +	− −	− −, − −	− −, − −
*R. commune*	D.1.1	Wall barley	Switzerland	2004	+ +	− −	− −, − −	− −, − −
*R. commune*	E.1.2	Wall barley	Switzerland	2004	+ +	− −	− −, − −	− −, − −
*R. commune*	2lm11	Italian ryegrass	Shropshire, UK	2011	+ +	− −	− −,++	− −, − −
*R. commune*	5lm11	Italian ryegrass	Shropshire, UK	2011	+ +	− −	− −, − +	− −, − −
*R. orthosporum*	57dg09	Cocksfoot	Aberystwyth, UK	2009	− −	+ +	− −, − −	− −, − −
*R. orthosporum*	59dg09	Cocksfoot	Aberystwyth, UK	2009	− −	− −	− −, − −	− −, − −
*R. orthosporum*	RsCH04 Bär A.1.1.3	Cocksfoot	Switzerland	2004	− −	+ +	− −, − −	− −, − −
*R. orthosporum*	RS04CG-BAR-A.1.1.3	Cocksfoot	Switzerland	2004	− −	+ +	− −, − −	− −, − −
*R. orthosporum*	RS04CG-BAR-A.1.1.4	Cocksfoot	Switzerland	2004	− −	+ +	− −, − −	− −, − −
*R. lolii*	6lm11	Italian ryegrass	Aberystwyth, UK	2011	− −	− −	+ −, − −	− +, − −
*R. lolii*	9lm11	Italian ryegrass	Aberystwyth, UK	2011	− −	− −	− −,+−	+ −, − −
*R. lolii*	10lm11	Italian ryegrass	Aberystwyth, UK	2011	− −	− −	− +,+−	+ −, − −
*R. lolii*	21lm11	Italian ryegrass	Shropshire, UK	2011	− −	− −	− +,++	− −, − −
*R. lolii*	22lm11	Italian ryegrass	Shropshire, UK	2011	− −	− −	+ −, − +	− +, − −
*R. lolii*	11lp11	Perennial ryegrass	Aberystwyth, UK	2011	− −	− −	+ −,+−	− +, − +
*R. lolii*	13lp11	Perennial ryegrass	Aberystwyth, UK	2011	− −	− −	− −, − −	− −,++
*R. lolii*	15lp11	Perennial ryegrass	Shropshire, UK	2011	− −	− −	− −, − −	− +, − −
*R. lolii*	16lp11	Perennial ryegrass	Surrey, UK	2011	− −	− −	− −, − −	− −,++
*R. lolii*	20lp11	Perennial ryegrass	Hertfordshire, UK	2011	− −	− −	− −,+−	− −, − +
n/a	Water control	n/a^f^	n/a	n/a	− −	− −	− −, − −	− −, − −

Leaf blotch disease symptoms developed at 23 days post inoculation when isolates of *Rhynchosporium commune*, *R. orthosporum* or *R. lolii* were cross-inoculated onto different hosts under controlled environment conditions.

a Whole plants scored for presence (+) or absence (−) of leaf blotch lesions;

b Scores for replicate plants are given;

c Plants 14-days-old at time of inoculation;

d Plants 35-days-old at time of inoculation;

e Plants 35- or 40- days-old at time of inoculation (results separated by a comma);

f n/a, not applicable.

All plants were grown and inoculated under controlled environment conditions, with a constant temperature of 15°C and a relative humidity of 70%. There was a 12 hour photoperiod with available light set at 700 µmol m^2^/sec. Seeds were sown in Rothamsted prescription mix compost (Petersfield Products, UK) at approximately 2 cm depth. Before inoculation, barley (cv. Optic) (14-day-old plants; seed supplied by the Rothamsted farm), cocksfoot (35-day-old plants) and both Italian and perennial ryegrass (35- or 40-day-old plants; seeds supplied by Herbiseed Ltd., UK) plants were grown individually in pots (4 cm×4 cm).

Immediately before inoculation, Falcon tubes containing *Rhynchosporium* inoculum were removed from storage at −20°C and allowed to defrost. Inoculum of each isolate was then transferred to a separate 20 ml plastic spray bottle. Conidial suspensions of each isolate were then sprayed onto two replicate whole plants of each graminaceous host, using a completely randomised design, until leaves were evenly coated with fine droplets. Plants were then maintained in a humid environment for 48 h by sealing them in 650–800 mm polyethylene bags (VWR International Ltd, UK). After this time, the top and bottom of each polyethylene bag were removed so that plants remained isolated but open to ambient relative humidity. In the experiments, sterile distilled water (containing 0.01% Tween 80) was used as a negative control. At 23 days post inoculation (dpi), whole plants were assessed for the presence or absence of typical leaf blotch lesions.

Barley and Italian ryegrass leaves that had been inoculated with two isolates of *R. commune* (2lm11 and 5lm11) that had produced typical leaf blotch lesions on both hosts (or with sterile distilled water, no lesions) were also examined in duplicate by scanning electron microscopy (SEM) (21 or 28 dpi as required, see Figure 4 legend).

### Colonisation Strategy

Plants of barley, rye, triticale, cocksfoot and Italian ryegrass were grown and inoculated under the controlled environment conditions for host range testing, except that leaves of the first three hosts were all 14-days-old at the time of inoculation and only the second fully expanded leaves were inoculated. Barley leaves (cv. Sumo) were inoculated with an isolate of *R. commune* (53hv09), rye leaves with an isolate of *R. secalis* (RS99CH1 H10B), triticale leaves with an isolate of *R. secalis* (I-1a), cocksfoot leaves with an isolate of *R. orthosporum* (RsCH04 Bär A.1.1.3) and Italian ryegrass leaves inoculated with an isolate originally collected from this host (9lm11) (see Table 2 for isolate details). Leaves of all of these graminaceous hosts with typical leaf blotch lesions were examined using SEM (14–30 dpi as required; see Figure 5 legend). In addition, couch-grass leaves colonised by *R. agropyri* and displaying typical leaf blotch lesions were also examined by SEM. However, it had not been possible to produce leaf blotch lesions by inoculating couch-grass with *R. agropyri* in controlled environments, despite the use of isolates from different geographical origins to inoculate couch grass plants of different ages. Therefore couch-grass leaf specimens colonised by *R. agropyri* were collected from the field in Hertfordshire (UK) in April 2010 and used for examination.

**Table 2:** Validation of species-specific diagnostic tests for five *Rhynchosporium* species.

Species	Isolate code	Host	Geographical origin	Collected	*R. commune* (LinA-F/R)	*R. agropyri* (RA6-F/R)	*R. secalis* (RS25-F/R)	*R. orthosporum*/*R. lolii* (2RO-F/R)	*R. lolii* (ERIC2/BOXAIR)
*R. commune*	788	Barley	France	1997	+	−	−		
*R. commune*	K1124	Barley	UK	Unknown	+	−	−		−
*R. commune*	QUB 12-3	Barley	Northern Ireland, UK	Unknown	+	−	−		−
*R. commune*	OSA 28-2-2	Barley	Hertfordshire, UK	2002	+	−	−		−
*R. commune*	FI12-63	Barley	Finland	1996	+	−	−		
*R. commune*	RS 219	Barley	UK	2004	+	−	−		
*R. commune*	QUB 9-10	Barley	Northern Ireland, UK	Unknown	+	−	−		
*R. commune*	R.s. 2310 4.2	Barley	France	2008	+	−	−		
*R. commune*	R.s. 2318 4.2	Barley	France	2008	+	−	−		−
*R. commune*	19hv09	Barley	Hertfordshire, UK	2009	+	−	−	−	−
*R. commune*	53hv09	Barley	Hertfordshire, UK	2009	+	−	−	−	−
*R. commune*	73hv09	Barley	Hertfordshire, UK	2009	+	−	−		−
*R. commune*	UK7	Barley	Aberystwyth, UK	Unknown	+	−	−	−	
*R. commune*	D.1.1	Wall barley	Switzerland	2004	+	−	−		−
*R. commune*	E.1.2	Wall barley	Switzerland	2004	+	−	−		−
*R. commune*	2lm11	Italian ryegrass	Shropshire, UK	2011	+	−	−	−	−
*R. commune*	5lm11	Italian ryegrass	Shropshire, UK	2011	+	−	−	−	−
*R. agropyri*	RS04CG-RAC-A.4.3	Couch-grass	Switzerland	2004	−	+	−		−
*R. agropyri*	RS04CG-RAC-A.5.2	Couch-grass	Switzerland	2004	−	+	−	−	−
*R. agropyri*	RS04CG-RAC-A.6.1	Couch-grass	Switzerland	2004	−	+	−	−	−
*R. agropyri*	1ar10	Couch-grass	Surrey, UK	2010	−	+	−		−
*R. agropyri*	2ar10	Couch-grass	Surrey, UK	2010	−	+	−	−	−
*R. agropyri*	3ar10	Couch-grass	Surrey, UK	2010	−	+	−	−	
*R. agropyri*	6ar10	Couch-grass	Cluj-Napoca, Romania	2010	−	+	−	−	−
*R. agropyri*	7ar10	Couch-grass	Timisoara, Romania	2010	−	+	−	−	−
*R. agropyri*	8ar10	Couch-grass	Nottingham, UK	2010	−	+	−	−	−
*R. agropyri*	9ar10	Couch-grass	Nottingham, UK	2010	−	+	−		−
*R. agropyri*	10ar10	Couch-grass	Nottingham, UK	2010	−	+	−	−	
*R. agropyri*	11ar10	Couch-grass	Nottingham, UK	2010	−	+	−	−	
*R. secalis*	RS02CH4-2a1	Rye	Switzerland	2002	−	−	+	−	−
*R. secalis*	RS02CH4-4b1	Rye	Switzerland	2002	−	−	+	−	−
*R. secalis*	RS02CH4-5a1	Rye	Switzerland	2002	−	−	+	−	−
*R. secalis*	Rs02CH4-6a.1	Rye	Switzerland	2002	−	−	+		−
*R. secalis*	RS99CH1-H10B	Rye	Switzerland	1999	−	−	+		−
*R. secalis*	RS02CH4-13a1	Rye	Switzerland	2002	−	−	+	−	−
*R. secalis*	RS02CH4-14a1	Rye	Switzerland	2002	−	−	+	−	
*R. secalis*	8.4	Rye	Russia	2003	−	−	+		−
*R. secalis*	6.2	Rye	Russia	2003	−	−	+	−	−
*R. secalis*	1E7a	Rye	Switzerland	1999	−	−	+	−	−
*R. secalis*	1B8	Rye	Switzerland	1999	−	−	+	−	−
*R. secalis*	1D4a	Rye	Switzerland	1999	−	−	+		−
*R. secalis*	I-1a	Triticale	Switzerland	2002	−	−	+	−	−
*R. secalis*	I-2a2	Triticale	Switzerland	2002	−	−	+	−	−
*R. secalis*	I-3a1	Triticale	Switzerland	2002	−	−	+		−
*R. orthosporum*	27dg09	Cocksfoot	Aberystwyth, UK	2009	−	−	−	+	−
*R. orthosporum*	51dg09	Cocksfoot	Aberystwyth, UK	2009				+	−
*R. orthosporum*	52dg09	Cocksfoot	Aberystwyth, UK	2009				+	−
*R. orthosporum*	57dg09	Cocksfoot	Aberystwyth, UK	2009	−	−	−	+	
*R. orthosporum*	59dg09	Cocksfoot	Aberystwyth, UK	2009	−	−	−	+	
*R. orthosporum*	RS04CG-BAR-A.1.1.3	Cocksfoot	Switzerland	2004	−	−	−	+	−
*R. orthosporum*	RS04CG-BAR-A.1.1.4	Cocksfoot	Switzerland	2004	−	−	−	+	−
*R. orthosporum*	RS04ITA D-6.1	Cocksfoot	Italy	2004	−	−	−	+	−
*R. orthosporum*	RS04ITA D-6.2	Cocksfoot	Italy	2004	−	−	−	+	−
*R. lolii*	1lm11	Italian ryegrass	Shropshire, UK	2011	−	−	−		+
*R. lolii*	3lm11	Italian ryegrass	Shropshire, UK	2011	−	−	−	+	+
*R. lolii*	4lm11	Italian ryegrass	Shropshire, UK	2011	−	−	−	+	+
*R. lolii*	6lm11	Italian ryegrass	Aberystwyth, UK	2011	−	−	−		+
*R. lolii*	7lm11	Italian ryegrass	Aberystwyth, UK	2011	−	−	−	+	+
*R. lolii*	8lm11	Italian ryegrass	Aberystwyth, UK	2011	−	−	−	+	+
*R. lolii*	9lm11	Italian ryegrass	Aberystwyth, UK	2011	−	−	−	+	
*R. lolii*	10lm11	Italian ryegrass	Aberystwyth, UK	2011	−	−	−		+
*R. lolii*	21lm11	Italian ryegrass	Shropshire, UK	2011	−	−	−		+
*R. lolii*	22lm11	Italian ryegrass	Shropshire, UK	2011	−	−	−		+
*R. lolii*	11lp11	Perennial ryegrass	Aberystwyth, UK	2011	−	−	−		
*R. lolii*	12lp11	Perennial ryegrass	Aberystwyth, UK	2011	−	−	−	+	+
*R. lolii*	13lp11	Perennial ryegrass	Aberystwyth, UK	2011	−	−	−	+	+
*R. lolii*	14lp11	Perennial ryegrass	Aberystwyth, UK	2011	−	−	−	+	+
*R. lolii*	15lp11	Perennial ryegrass	Shropshire, UK	2011	−	−	−	+	+
*R. lolii*	16lp11	Perennial ryegrass	Surrey, UK	2011	−	−	−		
*R. lolii*	17lp11	Perennial ryegrass	Hertfordshire, UK	2011	−	−	−	+	+
*R. lolii*	18lp11	Perennial ryegrass	Hertfordshire, UK	2011	−	−	−	+	+
*R. lolii*	20lp11	Perennial ryegrass	Hertfordshire, UK	2011	−	−	−	+	
*Leptosphaeria maculans*	LMA1	Oilseed rape	Unknown	Unknown	−	−	−	−	n/a
*L. maculans*	LMA5	Oilseed rape	Unknown	Unknown	−	−	−	−	n/a
*Pyrenopeziza brassicae*	PbCRA	Oilseed rape	UK	1988	−	−	−	−	n/a
*P. brassicae*	PbCRB	Oilseed rape	UK	1988	−	−	−	−	n/a
*Sclerotinia sclerotiorum*	SSGFRII	Oilseed rape	Unknown	Unknown	−	−	−	−	n/a
*Fusarium graminearum*	602.10	Wheat	Unknown	Unknown	−	−	−	−	n/a
*F. culmorum*	UK 99	Wheat	Unknown	Unknown	−	−	−	−	n/a
Host plant DNA^a^	n/a^b^	Barley	n/a	n/a	−	−	−	−	n/a
Host plant DNA^a^	n/a	Rye	n/a	n/a	−	−	−	−	n/a
Host plant DNA^a^	n/a	Cocksfoot	n/a	n/a				−	n/a

All the diagnostic tests detected only the predicted *Rhynchosporium* species when tested against a range of fungal isolates and plant DNA. PCR using primer pair LinA-F/R produced an amplicon of 145-bp specific for *R. commune* isolates; RA6-F/R produced an amplicon of 461-bp specific for *R. agropyri* isolates; RS25-F/R produced an amplicon of 1240-bp specific for *R. secalis* isolates; 2RO-F/R produced an amplicon of 277-bp specific for both *R. orthosporum* and *R. lolii* isolates; rep-PCR genomic fingerprinting using primers ERIC2/BOXA1R produced an amplicon of ∼400-bp specific for isolates of *R. lolii*.

a Barley (cv. Sumo), rye or cocksfoot DNA extracted from leaves of healthy seedlings (no leaf blotch lesions present) grown under controlled environment conditions was confirmed to be free of detectable *Rhynchosporium* DNA using the quantitative PCR assay of Fountaine *et al*. [30] (data not shown);

b n/a = not applicable.

For SEM, leaves were collected and placed in individual 9 cm diameter Petri dishes, which were lined with moist filter paper (Whatman No. 8) and sealed with a single layer of parafilm. Pieces of leaves, approximately 5 mm×5 mm, were cut out using a sterile razor blade. These were immediately mounted onto aluminium cryo-stubs using a smear of Tissue-Tek © O.C.T. compound (Sakura Finetek, USA) and plunged into pre-slushed liquid nitrogen (−280°C) to freeze them. The samples were then transferred under vacuum to the Alto 2100 (Gatan, UK) cryo-chamber stage, with the temperature maintained at −180°C. Sublimation of any contaminating ice and gold coating (10 nm thickness) was done in this chamber. Samples were then transferred to the stage of the scanning electron microscope (JSM 6360 LVSEM, Jeol, UK) with the temperature maintained at −150°C for examination and imaging using the on-board software (Jeol, UK).

### Species-specific PCR Diagnostic Tests

Four PCR-based diagnostic tests, relying on either sequence alignments of known genetic loci (ITS and beta-tubulin) and non-coding nuclear RFLP loci (pRS52; [27]) or using RAPD-PCR derived sequence, were developed to both detect and distinguish between isolates of (i) *R. commune* (including two isolates from Italian ryegrass), (ii) *R. agropyri*, (iii) *R. secalis*, (iv) *R. orthosporum* and all other *Rhynchosporium* isolates obtained from ryegrasses. Primers (for all sequences see Table S2) were designed using Vector NTI software (Invitrogen, USA). All PCR reactions were set up on ice (∼0°C) and used a PTC-100 programmable thermal controller (MJ Research, USA); all testing using these diagnostic tests included no template controls. PCR products (10 µl) were separated by gel electrophoresis in 1×TBE buffer on 1% agarose gels. Gels were either stained with ethidium bromide solution as described previously in the text or incorporated with ethidium bromide (0.5 µg/ml) during preparation. Amplicons were viewed on a transilluminator and digital images recorded (Gene Genius Bio Imaging System, Syngene, Synoptics Ltd, UK).

*R. commune*-specific primers (LinA-F/R) were designed based on alignments of partial sequences of the ITS region and were predicted to produce a 145-bp amplicon specific only for template DNA of this species. PCR was carried out in 20 µl reaction volumes, each containing 10 µl RedTaq ReadyMix (2×concentrate, Sigma Aldrich, UK), 2 µl each of both primers (1 pmol µl^−1^ stock), 5 µl of sterile distilled water and 1 µl of template DNA (10 ng). Reaction conditions were as follows; 35 cycles of 95°C for 1 min, 66°C for 1 min, 72°C for 1 min before a final elongation step of 72°C for 5 min, with a final hold at 4°C.

*R. agropyri-*specific primers (RA6-F/R) were designed based on alignments of pRS52 sequences [27] and were predicted to produce a 461-bp amplicon specific only for template DNA of this species. PCR was carried out in 25 µl reaction volumes, each containing 12.5 µl of RedTaq ReadyMix (2×concentrate, Sigma Aldrich, UK), 1 µl each of both primers (5 pmol µl^−1^ stock), 9.5 µl of sterile distilled water and 1 µl of template DNA (10 ng). Reaction conditions were as follows; 35 cycles of 94°C for 1 min, 55°C for 2 min and 72°C for 2 min before a final elongation step of 72°C for 5 min, with a final hold at 4°C.

*R. secalis-*specific primers were developed as sequenced characterised amplified region (SCAR) markers [28]. An amplicon of ∼1240-bp was produced specifically for isolates of *R. secalis* in RAPD-PCR testing with primer OPW-05. This amplicon was purified from an agarose gel using a QiaQuick gel extraction kit (Qiagen, UK) and cloned using a Strataclone PCR cloning kit (Agilent Technologies, UK). Plasmids with inserts of the correct size were then purified from cultures using the Fermentas GeneJet plasmid prep kit (Fermentas, UK) and sent to Eurofins MWG Operon for bi-directional sequencing using an ABI 3730XL machine. Resulting sequence data were then used to develop SCAR-PCR primers by extending the original 10-bp RAPD primer sequence into the flanking regions. These *R. secalis*-specific primers (RS25-F/R) were predicted to produce a 1240-bp amplicon specific only for template DNA of this species. PCR was carried out in 25 µl reaction volumes, each containing 12.5 µl RedTaq ReadyMix (2×concentrate, Sigma Aldrich, UK), 1 µl each of both primers (1 pmol µl^−1^ stock), 9.5 µl of sterile distilled water and 1 µl of template DNA (10 ng). Reaction conditions were as follows; 35 cycles of 94°C for 1 min, 57°C for 2 min, 72°C for 2 min before a final elongation step of 72°C for 5 min, with a final hold at 4°C.

Primers specific for *R. orthosporum* and most *Rhynchosporium* isolates from ryegrasses (2RO-F/R) were designed using alignments of partial sequences of the beta-tubulin region and were predicted to produce a 277-bp amplicon specific for template DNA of only these two species. PCR was carried out in 25 µl reaction volumes, each containing 12.5 µl RedTaq ReadyMix (2×concentrate), 1 µl each of both primers (1 pmol µl^−1^ stock), 9.5 µl of sterile distilled water and 1 µl of template DNA (1 ng). Reaction conditions were as follows; 35 cycles of 94°C for 1 min, 52°C for 1 min, 72°C for 1 min before a final elongation step of 72°C for 5 min, with a final hold at 4°C.

The specificity of all four diagnostic tests was evaluated by screening them against template DNA from a collection of *Rhynchosporium* isolates (Table 2). Specificity of the four tests was further confirmed by screening against template DNA of other crop pathogens, including the closely related *Pyrenopeziza brassicae* [29], and different plant hosts (e.g. barley) by PCR with the addition of the appropriate DNA template (in all cases, 10 ng template DNA was added; Table 2). In addition, the sensitivities of the four diagnostic tests were evaluated by screening against a total of 25 ng of mixed template DNA, with different amounts of DNA of the respective *Rhynchosporium* species (10 ng, 5 ng, 1 ng, 100 pg, 1 pg, 0 pg) used in a background of corresponding healthy host plant DNA (15 ng, 20 ng, 24 ng, 24.9 ng, 24.99 ng, 25 ng, respectively; confirmed to be free of detectable *Rhynchosporium* DNA using the quantitative PCR assay of Fountaine *et al*. [30] (data not shown). However, in the 20 or 25 µl reaction volume, as appropriate, 2 µl of template DNA was included by reducing by 1 µl the volume of sterile distilled water. The *R. commune* test was evaluated using isolate UK7 [31] in a background of healthy barley (cv. Sumo) plant template DNA, the *R. agropyri* test evaluated using isolate 7ar10 in a background of healthy barley (cv. Sumo) plant template DNA, the *R. secalis* test evaluated using isolate I-1a in a background of healthy rye plant template DNA and the *R. orthosporum*/most isolates from ryegrasses test evaluated using isolate 57dg09 in a background of healthy cocksfoot plant template DNA.

Finally, a fifth PCR-based test, based on data obtained from the rep-PCR genomic fingerprinting, was developed to distinguish between confirmed (using primer pair 2RO-F/R) isolates of *R. orthosporum* and most isolates from ryegrasses. Rep-PCR genomic fingerprinting using primer pair ERIC2/BOXA1R against a collection of 71 *Rhynchosporium* isolates was carried out as described previously, with these primers predicted to produce an ∼400-bp amplicon for most isolates from ryegrasses but not for isolates of *R. orthosporum.*

### Nomenclature

The electronic version of this article in Portable Document Format (PDF) is a work with an ISSN or ISBN number that will represent a published work according to the International Code of Nomenclature for algae, fungi, and plants and hence the new names contained in the electronic publication of a *PLOS ONE* article are effectively published under that Code from the electronic edition alone, so there is no longer any need to provide printed copies.

In addition, the new name contained in this work has been submitted to MycoBank (MB 803876) from where it will be made available to the Global Names Index. The unique MycoBank number can be resolved and the associated information viewed through any standard web browser by appending the MycoBank number contained in this publication to the prefix http://www.mycobank.org/mb/. The online version of this work is archived and available from the following digital repositories: PubMed Central, LOCKSS.

## Results

### DNA Fingerprint Analysis

**Figure 1:**
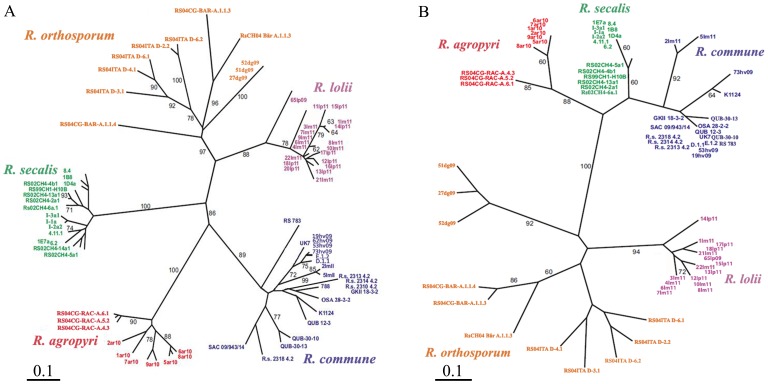
DNA fingerprinting methods distinguish between five *Rhynchosporium* species. (A) RAPD-PCR fingerprinting of 79 isolates using combined data from seven RAPD-PCR primers; (B) rep-PCR genomic fingerprinting of 71 isolates using combined data from two primer pairs (ERIC2/BOXA1R and ERICF/BOXA1R). Both unrooted trees were constructed by neighbour-joining analyses with branch lengths drawn to show genetic distance derived from Jaccard’s coefficient of band matching (scale bar: 0.1 = 10% genetic difference). Numbers at nodes indicate the percentage bootstrap support (based on 1000 re-samplings) for the groupings; only values (A) >60% and (B) >70% are shown, for clarity. Both fingerprinting methods discriminated between isolates of *R. commune* (blue), *R. agropyri* (red), *R. secalis* (green), *R. orthosporum* (yellow) and *R. lolii* (purple). Note that two isolates of *R. commune* (2lm11 and 5lm11) were collected from Italian ryegrass.

Both RAPD-PCR (Fig. 1A) and rep-PCR (Fig. 1B) methodologies could discriminate between isolates of *R. commune*, *R. agropyri*, *R. secalis* and *R. orthosporum* obtained from proximate geographical origins. Furthermore, in both analyses two distinct types of *Rhynchosporium* isolates were found on ryegrasses. Two isolates (2lm11 and 5lm11) collected from Italian ryegrass clustered within the main *R. commune* grouping, forming a terminal clade. However, all other isolates collected from both Italian and perennial ryegrasses formed a separate grouping with strong bootstrap support values (>85% for both DNA fingerprinting methods) distinct from all the other previously described species of *Rhynchosporium* (Figs 1A and 1B), consistent with the presence of a novel species. This grouping was tentatively named *Rhynchosporium lolii*, subject to further evidence of speciation that would allow a formal description (see below). *R. orthosporum* was the closest sister species to *R. lolii*, the latter of which showed an intermixing of isolates from both Italian and perennial ryegrasses.

### DNA Phylogeny and Times to the Most Recent Common Ancestor (TMRCA)

**Figure 2:**
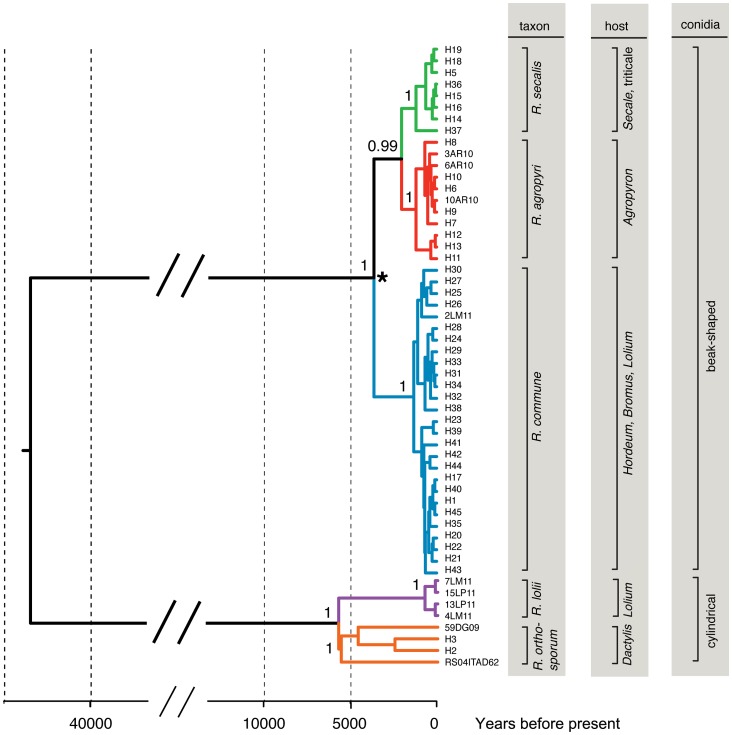
Multilocus phylogeny to determine the evolutionary relationships between five *Rhynchosporium* species. Phylogenetic analysis (maximum clade credibility tree) of combined partial sequences of the alpha-tubulin, beta-tubulin and internal transcribed spacer loci show consistent differences between *R. commune*, *R. agropyri*, *R. secalis*, *R. orthosporum* and *R. lolii*. Concatenated haplotype (H) sequences sourced from Zaffarano *et al*. [12] were combined with sequence data obtained from individual isolates in the present study. Posterior probabilities are indicated for major speciation nodes. The asterisk (*) indicates the calibration point used to infer absolute times (ybp; years before present) to the most recent common ancestor (TMRCA) for these *Rhynchosporium* species (see also Figure S1).

Bayesian phylogenetic analysis resulted in a tree topology with strongly supported clusters for all of the previously described *Rhynchosporium* species (Fig. 2). The phylogenetic analysis confirmed that two distinct *Rhynchosporium* species had been isolated from ryegrasses. Isolate 2lm11 was identified as *R. commune*, while isolates 4lm11, 7lm11, 13lp11 and 15lp11 grouped distinctly as the new *R. lolii* species. As with the previous DNA fingerprint analysis, *R. orthosporum* and *R. lolii* clustered as sister species, clearly separated from the three other species by a deep phylogenetic split. Estimates of TMRCA suggested a mean age of 736 years (160–1464 highest posterior density, HPD) for *R. lolii* and that it diverged from *R. orthosporum* ca. 5735 ybp (4335–7241). This TMRCA estimate for *R. lolii* overlaps largely with previous estimates for *R. secalis* (566–1922 ybp), *R. agropyri* (491–2023 ybp) and *R. commune* (775–1952 ybp) by Zaffarano *et al*. [12] (Figure S1).

### Conidial Morphology

**Figure 3:**
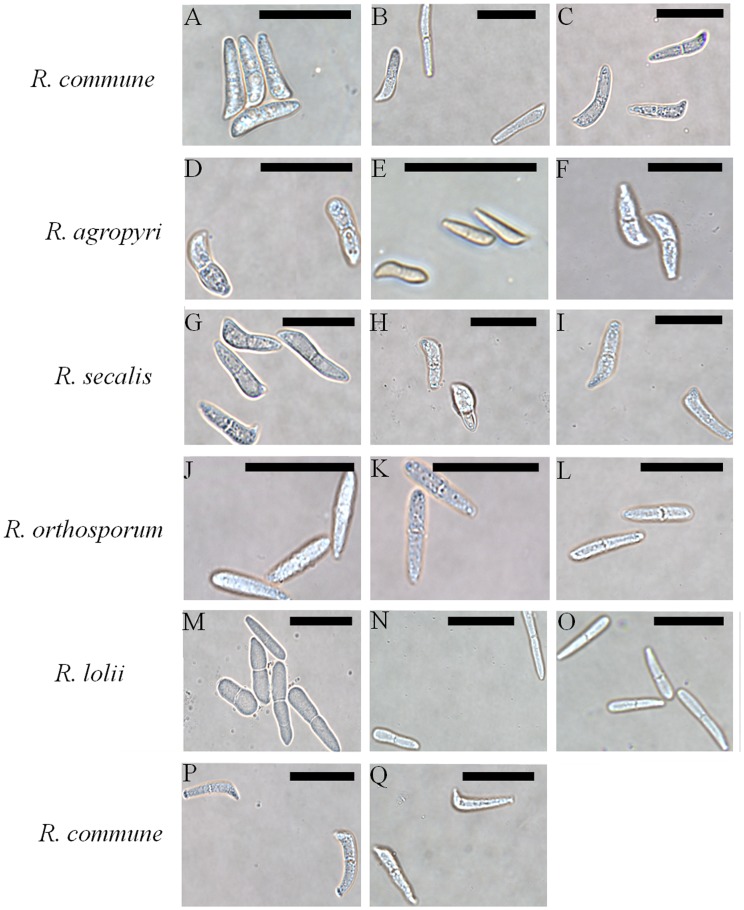
Five *Rhynchosporium* species divided into two groups by conidial shape. Isolates of *R. commune*, *R. agropyri* and *R. secalis* have beak-shaped conidia, while isolates of *R. orthosporum* and *R. lolii* have cylindrically-shaped conidia. Isolates shown are *R. commune* (collected from barley/wall barley) (A-C), *R. agropyri* (D-F), *R. secalis* (G-I), *R. orthosporum* (J-L), *R. lolii* (M-O) and *R. commune* (from Italian ryegrass) (P,Q). Isolates shown are (A-Q): 19hv09, D.1.1, E.1.2, 4ar10, 8ar10, Rs04CH Rac A.6.1, 1D4a, Rs02CH4-6a.1, I-3a1, 27dg09, 57dg09, RsCH04 Bär A.1.1.3, 12lp11, 20lp11, 22lm11, 2lm11, 5lm11. Scale bars are 20 *µm*.

Isolates could be divided into two distinct groups on the basis of conidial shape. Isolates of *R. commune* (including isolates 2lm11 and 5lm11 from Italian ryegrass), *R. agropyri* and *R. secalis* had beak-shaped conidia, while isolates of *R. orthosporum* and the new *R. lolii* species group from ryegrass had cylindrically-shaped conidia (Fig. 3). No statistically significant differences in conidial length were observed between isolates of *R. commune* (mean = 16.80 *µm*, standard error of the mean = 0.611), *R. agropyri* (15.41 *µm*, 0.576) or *R. secalis* (15.64 *µm*, 0.773) (*F*_2,35_ = 1.49, *P = *0.239). In addition, no significant differences in conidial width were observed between isolates of *R. commune* (2.95 *µm*, 0.152), *R. agropyri* (3.30 *µm*, 0.143) or *R. secalis* (3.19 *µm*, 0.192) (*F*_2,35_ = 1.48, *P = *0.241). In contrast, statistically significant differences in conidial length were observed between isolates of *R. orthosporum* (16.41 *µm*, 0.611) and *R. lolii* (19.36 *µm*, 0.546); (*F*_1,35_ = 12.09, *P*<0.01). However, no significant differences in conidial width were observed between isolates of *R. orthosporum* (2.92 *µm*, 0.152) and *R. lolii* (2.77 *µm*, 0.136) (*F*_1,35_ = 0.53, *P = *0.473). This differentiation in conidial morphology between the two groups, i.e. cylindrical vs. beak-shaped, was consistent with a deep phylogenetic split (see also Fig. 2).

### Host Range Testing

At 23 dpi under controlled environment conditions, five isolates of *R. commune* produced leaf blotch symptoms on barley (cv. Optic) but not on cocksfoot or ryegrasses. Likewise, four out of five isolates of *R. orthosporum* produced leaf blotch lesions only on cocksfoot but not on barley or ryegrasses (Table 1).

**Figure 4:**
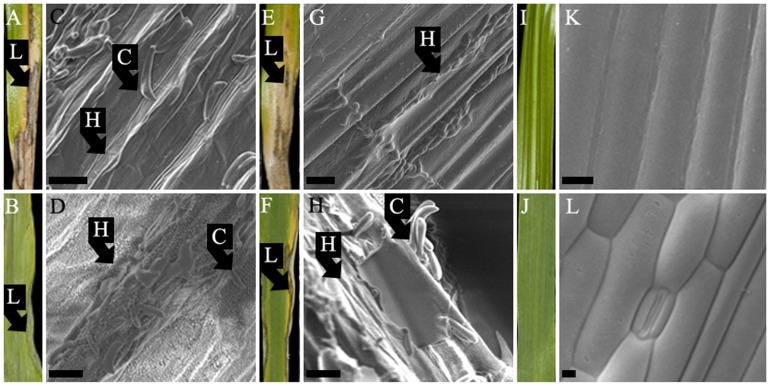
*R. commune* isolates that cause leaf blotch lesions on both Italian ryegrass and barley. Isolate 2lm11 caused lesions **(L)** when inoculated onto (A) Italian ryegrass or (B) barley (cv. Optic) leaves; scanning electron microscopic (SEM) examination of these hosts (C, D) showed sub-cuticular hyphae **(H)** and sporulation with beak-shaped conidia **(C)** on both hosts. Isolate 5lm11 also caused lesions on both Italian ryegrass (E) and barley (F); SEM examination showed that the pathogen could colonise both hosts (G, H) and sporulation with beak-shaped conidia was observed on barley. Control leaves of Italian ryegrass and barley treated with water (I, J) did not develop leaf blotch symptoms and SEM examination (K, L) found no evidence for the presence of *R. commune*. Photographs of leaf symptoms were taken at 17 (B, F, J) and 24 (A, E, I) days post inoculation (dpi); electron micrographs were taken at 21 (D, H, L) and 28 (C, G, H) dpi. All leaf pieces were c. 4 cm long; scale bars on electron micrographs are 10 *µm*.

However, isolates collected from ryegrasses showed two distinct host range profiles (Table 1). Firstly, two isolates of *R. commune* collected from Italian ryegrass caused leaf blotch symptoms (Fig. 4A, B, E, F) on both barley (cv. Optic) and Italian ryegrass (although no disease symptoms developed on perennial ryegrass); by 23 dpi, both hosts showed extensive leaf blotch symptoms and total cell collapse was observed (Table 1). When examined with scanning electron microscopy, leaves of both barley (21 dpi) and Italian ryegrass (28 dpi) were extensively colonised by sub-cuticular hyphae (Fig. 4C, D, G, H). Moreover, beak-shaped conidia were observed (Fig. 4C, D, H) erupting through the leaf cuticle of both hosts (although no sporulation was observed on Italian ryegrass leaves inoculated with isolate 5lm11; Fig. 4G).

Secondly, ten isolates of *R. lolii* obtained from either Italian or perennial ryegrass produced leaf blotch lesions only on ryegrasses and not on barley or cocksfoot (Table 1). Some isolates of *R. lolii* produced leaf blotch symptoms on both Italian and perennial ryegrass; therefore, there was no evidence for specialisation between these hosts.

### Colonisation Strategy

**Figure 5:**
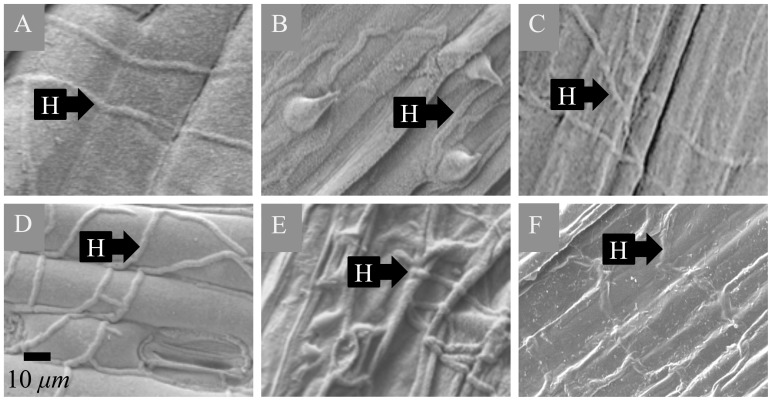
Five *Rhynchosporium* species colonise the same sub-cuticular niche in their hosts. Sub-cuticular hyphal **(H)** growth of (A) *R. commune* (isolate 53hv09) at 28 days post inoculation (dpi) on a leaf of barley (cv. Sumo); (B) *R. agropyri* on a leaf of couch-grass collected from the field (Hertfordshire, UK) in April 2010; (C) *R. secalis* (RS99CH1 H10B) at 30 dpi on a leaf of rye; (D) *R. secalis* isolate (I-1a) at 28 dpi on a leaf of triticale; (E) *R. orthosporum* (RsCH04 Bär A.1.1.3) at 14 dpi on a leaf of cocksfoot; (F) *R. lolii* (9lm11) at 28 dpi on a leaf of Italian ryegrass. Scale-bars on electron micrographs are 10 *µm*.

Scanning electron microscopy results showed that isolates of *R. commune* on barley (Fig. 5A), *R. agropyri* on couch-grass (Fig. 5B), *R. secalis* on rye (Fig. 5C), *R. secalis* on triticale (Fig. 5D), *R. orthosporum* on cocksfoot (Fig. 5E) and *R. lolii* on Italian ryegrass (Fig. 5F) all colonised their respective hosts in a similar manner. In all of these pathosystems, clear evidence was found for extracellular growth of the pathogen, with extensive, branching hyphae colonising the sub-cuticular area of the host leaf tissue. In addition, sub-cuticular hyphal growth was also observed on most of these hosts on areas of leaf tissue with no visible leaf blotch symptoms, i.e. there was evidence of asymptomatic colonisation surrounding lesions (data not shown).

### Species-specific PCR Diagnostic Tests

**Figure 6:**
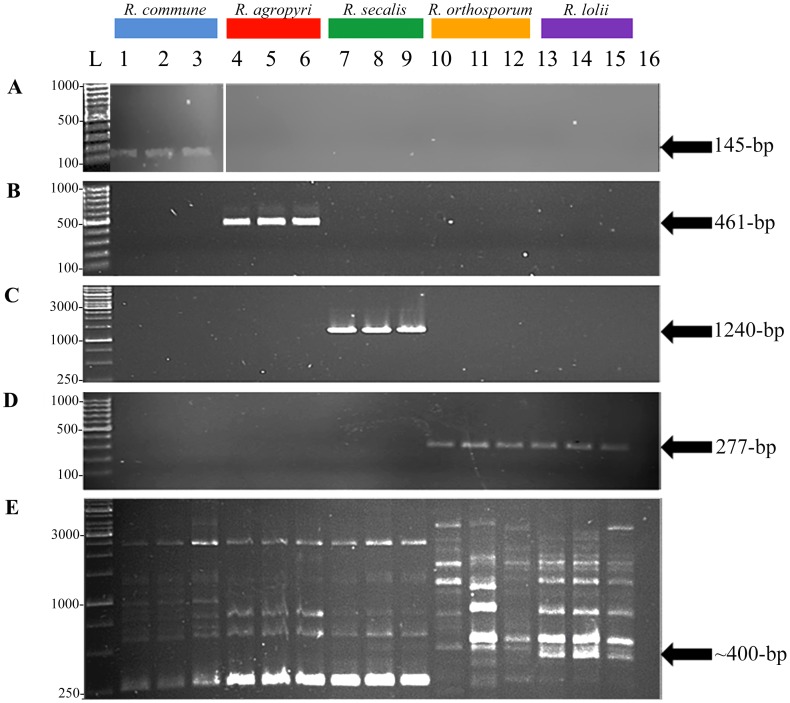
PCR-based diagnostic tests to distinguish between five *Rhynchosporium* species. Five primer pairs (LinA-F/R; RA6-F/R; RS25-F/R; 2RO-F/R; ERIC2/BOXA1R) were tested with representative isolates of *R. commune* (lanes 1–3; K1124, QUB 12-3, OSA 28-2-2), *R. agropyri* (lanes 4–6; RS04CG-RAC-A.6.1, 6ar10, 10ar10), *R. secalis* (lanes 7–9; RS99CH1 H10B, E7a, I-1a), *R. orthosporum* (lanes 10–13; 27dg09, RS04CG-BAR-A.1.1.4, RS04ITA D-4.1) or *R. lolii* (lanes 13–15; 1lm11, 9lm11 and 18lp11). (A) LinA-F/R produced a 145-base pair (bp) amplicon specific for *R. commune* isolates; (B) RA6-F/R produced a 461-bp amplicon specific for *R. agropyri* isolates; (C) RS25-F/R produced a 1240-bp amplicon specific for *R. secalis* isolates; (D) 2ROR-F/R produced a 277-bp amplicon specific for both *R. orthosporum* and *R. lolii* isolates; use of rep-PCR genomic fingerprinting primers ERIC2/BOXA1R produced a ∼400-bp amplicon specific only for isolates of *R. lolii*. Different isolates are displayed in (B, C) lanes 1–3: FI12-63, QUB 9-10, 2lm11; (D) lanes 1–15∶19hv09, UK7, 2lm11, 10ar10, 6ar10, RS04CG-RAC-A.6.1, RS02CH4-4b1, RS02CH4-14a1, 6.2, 27dg09, RS04CG-BAR-A.1.1.4, RS04ITA D-4.1, 9lm11, 14lp11 and 18lp11; (E) lanes 3, 6 and 14∶2lm11, 1ar10 and 8lm11, respectively. Note that in (A) lanes 1–3 have been inserted from a different gel image. Lane labelled ‘L’ contains a 100-bp ladder (A, B, D) or a 1-kilobase ladder (C, E) (both Fermentas, UK); lane 16 is a no template control.

Species-specific endpoint PCR diagnostic tests were developed to detect and distinguish between isolates of (i) *R. commune*, (ii) *R. agropyri*, (iii) *R. secalis* and (iv) *R. orthosporum/R. lolii.* Multiple alignments of known genetic loci were made (or RAPD-PCR derived sequence used) and a series of different primer pairs were designed with the theoretical ability to discriminate between species due to sequence divergence at the 3′ end and/or within the primers. Many of these primer pairs failed to discriminate sufficiently between species, with false positives produced in some reactions (data not shown). However, a subset of these primer pairs consistently discriminated between the *Rhynchosporium* species. These diagnostic primers were evaluated by screening against a set of *Rhynchosporium* isolates representing the different species from proximate geographical origins (Table 2). The *R. commune* diagnostic test (primer set LinA-F/R) unambiguously produced the predicted amplicon of 145-bp (Fig. 6A) only with 10 ng template DNA from *R. commune* isolates. However, it is noted that an extremely faint and ambiguous PCR signal was produced for a single isolate of *R. agropyri* (RS04CG-RAC-A.4.3); no signal was detected when the PCR was repeated with only 34 cycles (data not shown). The *R. agropyri* test (RA6-F/R) produced the predicted 461-bp (Fig. 6B) amplicon only with 10 ng template DNA from *R. agropyri* isolates, while the *R. secalis* test (RS25-F/R) produced the predicted 1240-bp (Fig. 6C) amplicon only with 10 ng template DNA from *R. secalis* isolates. The *R. orthosporum/R. lolii* test (2RO-F/R) produced the predicted 277-bp (Fig. 6D) amplicon only with 1 ng template DNA from isolates of these two species; in addition, no amplicons were produced when this test was further screened against 10 ng template DNA from representative isolates of *R. commune*, *R. agropyri* and *R. secalis* (data not shown). None of the four species-specific diagnostic tests detected 10 ng template DNA of either plant host or other fungal pathogens. These included five other pathogens of crops (Table 2), including the closely related *P. brassicae* [29].

Sensitivity testing, using mixed amounts of fungal/host plant DNA (25 ng template DNA in total), demonstrated that each of these four diagnostic tests were specific to their respective *Rhynchosporium* species in a background of host plant DNA, and that the sensitivity of each diagnostic test to the target species template DNA differed as follows; the *R. commune* test was by far the least sensitive test and required ∼2.5 ng, the *R. orthosporum/R. lolii* test required 1 ng and both the *R. agropyri* and *R. secalis* tests required 1 pg (data not shown).

Finally, the fifth rep-PCR genomic fingerprinting based diagnostic (using primer pair ERIC2/BOXA1R) was also developed; this produced an amplicon of ∼400-bp for isolates of *R. lolii* but not for isolates of *R. orthosporum* (Table 2; Fig. 6E). Therefore, when this test was used in combination with the *R. orthosporum/R. lolii* endpoint diagnostic test (Table 2; Fig 6D), these two species could readily be distinguished from each other.

### Taxonomy

Results described above provided clear evidence for the presence of a new species of *Rhynchosporium*, based on combined molecular, morphological and host specialisation data. Therefore, the new species, *Rhynchosporium lolii*, is now formally described:

*Rhynchosporium lolii* King, West, Brunner, Dyer and Fitt sp. nov. [urn:lsid:mycobank.org:names:803876] Etymology: Referring to the host genus, i.e. *Lolium*.

Type: UK: Shropshire: Newport, isolated from *Lolium perenne* leaves, May 2011, Collector: Kevin M. King; 15lp11 (IMI 502640– holotype (dried culture); CBS 135745 and IMI 502640– ex-holotype cultures).

*Rhynchosporium lolii* is genetically most closely related to *Rhynchosporium orthosporum*, and both species have conidia that are erect, cylindrically shaped and medianly septate. Conidia of *R. lolii* have a mean length of 19.36 *µm* and width of 2.77 *µm*; they are statistically significantly longer than those of *R. orthosporum*. The two species can be distinguished by the following fixed nucleotide differences between *R. lolii* and *R. orthosporum* (presented as the gene and the nucleotide characters fixed in *R. lolii* in parenthesis, based on numbering of the partial sequences deposited in GenBank in the present study); alpha-tubulin positions 85 (G), 100 (T), 499 (G); beta-tubulin position 309 (A). PCR amplification with primer pair 2RO-F/R produces an amplicon of 277 base pairs for both *R. lolii* and *R. orthosporum*, but amplification with rep-PCR genomic fingerprinting primer pair ERIC2/BOXA1R produces an amplicon of approximately 400 base pairs specifically for *R. lolii. R. lolii* causes leaf blotch lesions on ryegrass species but not on cocksfoot or barley.

## Discussion

This work provides the first conclusive evidence for the occurrence of both *R. commune* and a new species, *R. lolii*, on ryegrasses in the UK. Two *Rhynchosporium* isolates from Italian ryegrass (2lm11 and 5lm11) clustered within the main *R. commune* species group according to both DNA fingerprinting and sequence data. However, in both RAPD-PCR and rep-PCR genomic fingerprinting the two isolates formed a terminal clade with strong bootstrap support. It is unclear whether this was an artefact because these isolates originated from the same geographic location, or whether there was some genuine difference between *R. commune* isolates occurring on ryegrasses and those on barley.

In addition, the confirmed *R. commune* isolates from Italian ryegrass both had beak-shaped conidia and could colonise, cause leaf blotch symptoms and sporulate on both Italian ryegrass and barley (cv. Optic). These data support previous work by Wilkins [11], where some *Rhynchosporium* isolates were collected from Italian ryegrass that could cause disease symptoms on both this host and barley. However, it is noted that data in both the present and previous study [11] suggest that isolates of *R. commune* collected from barley do not appear to cause leaf blotch symptoms on Italian ryegrass. Nevertheless, we propose that the species description for *R. commune* [13] be amended to include Italian ryegrass as an additional host species.

The second species, *R. lolii*, occurred widely on ryegrasses in England and Wales. This new species was most closely related to *R. orthosporum* and these two sister species had cylindrically-shaped conidia, unlike other species of *Rhynchosporium*. However, all of the molecular approaches used revealed consistent differences between *R. lolii* and *R. orthosporum*. There was therefore sufficient phylogenetic evidence for the identification and description of the new species *R. lolii.* Such molecular phylogenetic evidence is now being used routinely to define new species of filamentous fungi and yeasts [32], [13], [33], [34], [35] because strongly supported molecular divergence between taxa, especially when based on multiple gene genealogies, indicates a lack of gene flow consistent with speciation events [36], [37], [38].

The new species *R. lolii* could also be distinguished on the basis of morphological differences, since it had cylindrically-shaped conidia that were typically longer than those of *R. orthosporum*. Furthermore, host range testing demonstrated that isolates of *R. lolii* caused leaf blotch symptoms only on ryegrass species whereas isolates of *R. orthosporum* caused leaf blotch symptoms only on cocksfoot. The occurrence of these host-specialised *Rhynchosporium* species is similar to that for other plant pathogen genera, for example *Zymoseptoria* and *Colletotrichum* that both include several species that are specialised to different wild and cultivated (cereal) grasses [34], [39].

Isolates of *R. orthosporum* and *R. lolii* from proximate geographical origins could also be distinguished by the new PCR-based diagnostic tests. The divergence of the two species was supported by results from a molecular clock model implemented in BEAST [25], which suggested that *R. lolii* diverged from *R. orthosporum ca*. 5735 ybp (range 4335–7241 ybp). Moreover, the TMRCA estimate of a mean age of 736 years (160–1464 HPD) for *R. lolii* largely overlaps with previous estimates for other *Rhynchosporium* species, suggesting independent speciation events during the same period [12].

This study has therefore clarified the identity of *Rhynchosporium* species occurring on ryegrasses, which are both economically important forage grasses and commonly occurring weeds of cereal crops throughout the world [11], [40], [15], [16]. These findings will be of practical use to both farmers and breeders, for example when considering the use of cocksfoot and ryegrass species as forage grasses [11]. Furthermore, these data suggest that ryegrasses could potentially be a reservoir of *R. commune* inoculum able to initiate barley leaf blotch epidemics. Similar roles for wild grasses as potential sources of inoculum have been suggested previously for the wheat pathogens *Pyrenophora tritici-repentis* (cause of tan spot), *Oculimacula yallundae* (synonym *Tapesia yallundae*, cause of eyespot) and *Colletotrichum* species (cause of anthracnose disease) [41], [42], [39]. Further work is now required to investigate the worldwide frequency and distribution of *R. commune* on ryegrass species.

This work has also demonstrated that all five *Rhynchosporium* species colonise their respective hosts in a very similar manner morphologically, with extensive hyphal growth observed in the sub-cuticular region of the leaves of all host species. It provides the first SEM evidence of morphological events relating to the colonisation strategy of *R. agropyri* on couch-grass, *R. secalis* on rye and triticale and *R. lolii* on Italian ryegrass. Few plant pathogenic fungi are known to grow in the sub-cuticular region, although other pathogens known to exploit this niche include *Diplocarpon rosae* (cause of blackspot) on roses (*Rosa* spp.) [43] and *P. brassicae* (cause of light leaf spot) on oilseed rape (*Brassica napus*) [44], [45]. Increased opportunities for horizontal gene transfer between these species due to occupation of the same sub-cuticular niche could have impacts on both disease emergence and metabolic capabilities [46].

Finally, the present study has developed the first endpoint PCR diagnostic tests that can directly detect and distinguish between isolates of *R. commune*, *R. agropyri*, *R. secali*s and *R*. *orthosporum* [14], [13]. However, the test developed for *R. orthosporum* also detected isolates of *R. lolii*; therefore an additional rep-PCR genomic fingerprinting based test was developed that distinguished these two sister species from each other. Rapid, simple and cheap diagnostic tests to distinguish between all these host-specialised *Rhynchosporium* species will benefit breeders, farmers and researchers because leaf blotch is a serious disease of barley crops across the world, especially in areas with cool temperate climates [1]. Moreover, leaf blotch is also an important disease of rye, triticale, cocksfoot and ryegrasses [8], [9], [10], [11]. These diagnostic tests may be of use in the detection of asymptomatic colonisation by these *Rhynchosporium* species on their respective hosts, such as has been observed on barley [47] and other grass hosts (present study). In addition, they will also complement PCR-based tests for other pathogens, such as *Ramularia collo-cygni* [48], an emerging pathogen of barley crops in northern Europe and New Zealand [49].

## Supporting Information

Figure S1 **Estimates of time to most recent common ancestor (TMRCA) for the five** ***Rhynchosporium*** **species.** Estimates were inferred from the phylogenetic reconstruction shown in Fig. 2. Indicated are mean (○) and 95% credibility intervals (vertical bars, i.e. highest posterior density, HPD).
(TIFF)

Table S1 ***Rhynchosporium*** **isolates used for DNA fingerprint testing and spore morphology measurements.**
(DOCX)

Table S2 **Primers used for DNA fingerprinting, amplification of gene loci or for discrimination between five** ***Rhynchosporium*** **species.**
(DOCX)

Table S3 **GenBank accession numbers for sequences obtained in the present study.**
(DOCX)

## References

[1] Zhan J, Fitt BDL, Pinnschmidt HO, Oxley SJP, Newton AC (2008) Resistance, epidemiology and sustainable management of *Rhynchosporium secalis* populations on barley. Plant Pathol 57: 1–14.

[2] Shipton WA, Boyd JR, Ali SM (1974) Scald of barley. Rev. Plant Pathol. 53: 839–861.

[3] HGCA website. Available: http://www.hgca.com/publications/documents/cropresearch/barley_growth_guide.pdf. Accessed: 22 Jul 2013.

[4] HGCA website. Available: http://publications.hgca.com/publications/documents/cropresearch/G44_-_HGCA_Barley_Disease_Management_Guide_2011_(Complete_version).pdf. Accessed: 22 Jul 2013.

[5] Avrova A, Knogge W (2012) *Rhynchosporium commune*: a persistent threat to barley cultivation. Mol Plant Pathol 13: 986–997. 22738626 10.1111/j.1364-3703.2012.00811.x PMC6638709

[6] Badr A, Müller K, Schäfer-Pregl R, El Rabey H, Effgen S, et al (2000) On the origin and domestication history of barley (*Hordeum vulgare*). Mol Biol Evol 17: 499–510. 10742042 10.1093/oxfordjournals.molbev.a026330

[7] Brunner PC, Schürch S, McDonald BA (2007) The origin and colonization history of the barley scald pathogen *Rhynchosporium secalis* . J Evol Biol 20: 1311–1321. 17584226 10.1111/j.1420-9101.2007.01347.x

[8] Brooks FT (1928) Observations on *Rhynchosporium secalis* (Oud.) Davis, leaf blotch of barley and rye. New Phytol 11: 215–219.

[9] Welty RE, Metzger RJ (1996) First report of scald of triticale caused by *Rhynchosporium secalis* in North America. Plant Dis 80: 1220–1223.

[10] Fernandez JP, Welty RE (1991) Histopathology of orchardgrass infected by *Rhynchosporium orthosporum* . Mycologia 83: 774–778.

[11] Wilkins P (1973) Infection of *Lolium multiflorum* with *Rhynchosporium* species. Plant Pathol 22: 107–111.

[12] Zaffarano PL, McDonald BA, Linde CC (2008) Rapid speciation following recent host shifts in the plant pathogenic fungus *Rhynchosporium* . Evolution 62: 1418–1436. 18384659 10.1111/j.1558-5646.2008.00390.x

[13] Zaffarano PL, McDonald BA, Linde CC (2011) Two new species of *Rhynchosporium* . Mycologia 103: 195–202. 20943529 10.3852/10-119

[14] Caldwell RM (1937) *Rhynchosporium* scald of barley, rye and other grasses. J Agric Res 55: 175–198.

[15] Charmet G, Balfourier F, Chatard V (1996) Taxonomic relationships and interspecific hybridization in the genus *Lolium* (grasses). Genet Resour Crop Evol 43: 319–327.

[16] Preston C, Wakelin AM, Dolman FC, Bostamam Y, Boutsalis P (2009) A decade of glyphosate-resistant *Lolium* around the world: mechanisms, genes, fitness, and agronomic management. Weed Sci 57: 435–441.

[17] Howlett SG, Cooke BM (1987) Scanning electron microscopy of sporulation in *Rhynchosporium secalis* . Trans Br Mycol Soc 88: 547–557.

[18] Lange BJ, Boyd WJR (1968) Preservation of fungal spores by drying on porcelain beads. Phytopathology 58: 1711–1712.

[19] Murtagh GJ, Dyer PS, McClure PC, Crittenden PD (1999) Use of randomly amplified polymorphic DNA markers as a tool to study variation in lichen-forming fungi. Lichenologist 31: 257–267.

[20] Hampl V, Pavlícek A, Flegr J (2001) Construction and bootstrap analysis of DNA fingerprinting-based phylogenetic trees with a freeware program FreeTree: Application to trichomonad parasites. Int J Syst Evol Microbiol 51: 731–735. 11411692 10.1099/00207713-51-3-731

[21] Page RDM (1996) TREEVIEW: An application to display phylogenetic trees on personal computers. Comput Appl Biosci 12: 357–358. 8902363 10.1093/bioinformatics/12.4.357

[22] Versalovic J, Koeuth T, Lupski JR (1991) Distribution of repetitive DNA sequences in eubacteria and application to fingerprinting of bacterial genomes. Nucleic Acids Res 19: 6823–6831. 1762913 10.1093/nar/19.24.6823 PMC329316

[23] Versalovic J, Schneider M, de Bruijn FJ, Lupski JR (1994) Genomic fingerprinting of bacteria using repetitive sequence based polymerase chain reaction. Methods Mol Cell Biol 5: 25–40.

[24] Hall TA (1999) BioEdit: a user-friendly biological sequence alignment editor and analysis program for Windows 95/98/NT. Nucleic Acids Symp Ser 41: 95–98.

[25] Drummond AJ, Rambaut A (2007) BEAST: Bayesian evolutionary analysis by sampling trees. BMC Evol Biol 7: 214. 17996036 10.1186/1471-2148-7-214 PMC2247476

[26] Payne RW, Murray DA, Harding SA, Baird DB, Soutar DM (2009) GenStat for Windows (12th Edition) Introduction. VSN International, Hemel Hempstead.

[27] Zaffarano PL, McDonald BA, Linde CC (2009) Phylogeographical analyses reveal global migration patterns of the barley scald pathogen *Rhynchosporium secalis* . Mol Ecol 18: 279–293. 19076278 10.1111/j.1365-294X.2008.04013.x

[28] Nicholson P, Rezanoor HN, Simpson DR, Joyce D (1997) Differentiation and quantification of the cereal eyespot fungi *Tapesia yallundae* and *Tapesia acuformis* using a PCR assay. Plant Pathol 46: 842–856.

[29] Goodwin SB (2002) The barley scald pathogen *Rhynchosporium secalis* is closely related to the discomycetes *Tapesia* and *Pyrenopeziza* . Mycol Res 106: 645–654.

[30] Fountaine JM, Shaw MW, Napier B, Ward E, Fraaije BA (2007) Application of real-time and multiplex polymerase chain reaction assays to study leaf blotch epidemics in barley. Phytopathology 97: 297–303. 18943648 10.1094/PHYTO-97-3-0297

[31] Lehnackers H, Knogge W (1990) Cytological studies on the infection of barley cultivars with known resistance genotypes by *Rhynchosporium secalis*. Canadian Journal of Botany, 68, 1953–1961.

[32] Fisher MC, Koenig GL, White TJ, Taylor JW (2002) Molecular and phenotypic description of *Coccidioides posadasii* sp. nov, previously recognized as the non-Californian population of *Coccidioides immitis* . Mycologia 94: 73–84. 21156479

[33] Holland SL, Dyer PS, Bond CJ, James SA, Roberts IN, et al (2011) *Candida argentea* sp. nov., a copper and silver resistant yeast species. Fungal Biol 115: 909–918. 21872188 10.1016/j.funbio.2011.07.004

[34] Stukenbrock EH, Quaedvlieg W, Javan-Nikhah M, Zala M, Crous PW, et al (2012) *Zymoseptoria ardabiliae* and *Z. pseudotritici*, two progenitor species of the septoria tritici leaf blotch fungus *Z. tritici* (synonym: *Mycosphaerella graminicola*). Mycologia 104: 1397–1407. 22675045 10.3852/11-374

[35] Barrs VR, van Doorn TM, Houbraken J, Kidd SE, Martin P, et al (2013) *Aspergillus felis* sp. nov., an emerging agent of invasive aspergillosis in humans, cats and dogs. PLoS One 8: e64871 doi:10.1371/journal.pone.0064871 23798996 10.1371/journal.pone.0064871 PMC3683053

[36] Taylor JW, Jacobson DJ, Kroken S, Kasuga T, Geiser DW, et al (2000) Phylogenetic species recognition and species concepts in fungi. Fungal Genet Biol 31: 21–31. 11118132 10.1006/fgbi.2000.1228

[37] Dettman JR, Jacobson DJ, Turner E, Pringle A, Taylor JW (2003) Reproductive isolation and phylogenetic divergence in *Neurospora* – comparing methods of species recognition in a model eukaryote. Evolution 57: 2721–2741. 14761052 10.1111/j.0014-3820.2003.tb01515.x

[38] Taylor JW, Turner E, Pringle A, Dettman J, Johannesson H (2006). Fungal species: thoughts on their recognition, maintenance and selection. In: Fungi in the Environment (Eds GM Gadd, SC Watkinson & PS Dyer), 313–339. Cambridge University Press, Cambridge.

[39] Crouch JA, Tredway LP, Clarke BB, Hillman BI (2009) Phylogenetic and population genetic divergence correspond with habitat for the pathogen *Colletotrichum cereale* and allied taxa across diverse grass communities. Mol Ecol 18: 123–135. 19076279 10.1111/j.1365-294X.2008.04008.x

[40] Lutman PJW, Storkey J, Martin H, Holland J (2009) Abundance of weeds in arable fields in southern England in 2007/08. Asp Appl Biol 91: 163–168.

[41] Kastelein P, Köhl J, Gerlagh M, Goossen-van de Geijn HM (2002) Inoculum sources of the tan spot fungus *Pyrenophora tritici-repentis* in The Netherlands. Meded Rijksuniv Gent Fak Landbouwkd Toegep Biol Wet 67: 257–267. 12701430

[42] Dyer PS, Bradshaw RE (2002) First report of apothecia of *Tapesia yallundae* occurring on the wild grass *Holcus lanatus* (Yorkshire fog) in New Zealand. Plant Pathol 51: 806.

[43] Gachomo EW, Dehne H-W, Steiner U (2009) Efficacy of triazoles and strobilurins in controlling black spot disease of roses caused by *Diplocarpon rosae* . Ann Appl Biol 154: 259–267.

[44] Boys EF, Roques SE, Ashby AM, Evans N, Latunde-Dada AO, et al (2007) Resistance to infection by stealth: *Brassica napus* (winter oilseed rape) and *Pyrenopeziza brassicae* (light leaf spot). Eur J Plant Pathol 118: 307–321.

[45] Boys EF, Roques SE, West JS, Werner CP, King GJ, et al (2012) Effects of *R* gene-mediated resistance in *Brassica napus* (oilseed rape) on asexual and sexual sporulation of *Pyrenopeziza brassicae* (light leaf spot). Plant Pathol 61: 543–554.

[46] Fitzpatrick DA (2012) Horizontal gene transfer in fungi. FEMS Microbiol Lett 329: 1–8. 22112233 10.1111/j.1574-6968.2011.02465.x

[47] Davis H, Fitt BDL (1990) Symptomless infection of *Rhynchosporium secalis* on leaves of winter barley. Mycol Res 94: 557–560.

[48] Taylor JMG, Paterson LJ, Havis ND (2010) A quantitative real-time PCR assay for the detection of *Ramularia collo-cygni* from barley (*Hordeum vulgare)* . Lett Appl Microbiol 50: 493–499. 20337932 10.1111/j.1472-765X.2010.02826.x

[49] Walters DR, Havis ND, Oxley SJ (2008) *Ramularia collo-cygni*: the biology of an emerging pathogen of barley. FEMS Microbiol Lett 279: 1–7. 18070071 10.1111/j.1574-6968.2007.00986.x

