# Supplementary figures and images for: Evolutionary Relationships between Rhynchosporium lolii sp. nov. and Other Rhynchosporium Species on Grasses
Source: PLoS One. 2013 Oct 16;8(10):e72536. doi: 10.1371/journal.pone.0072536 (PMC3797698; doi:10.1371/journal.pone.0072536)

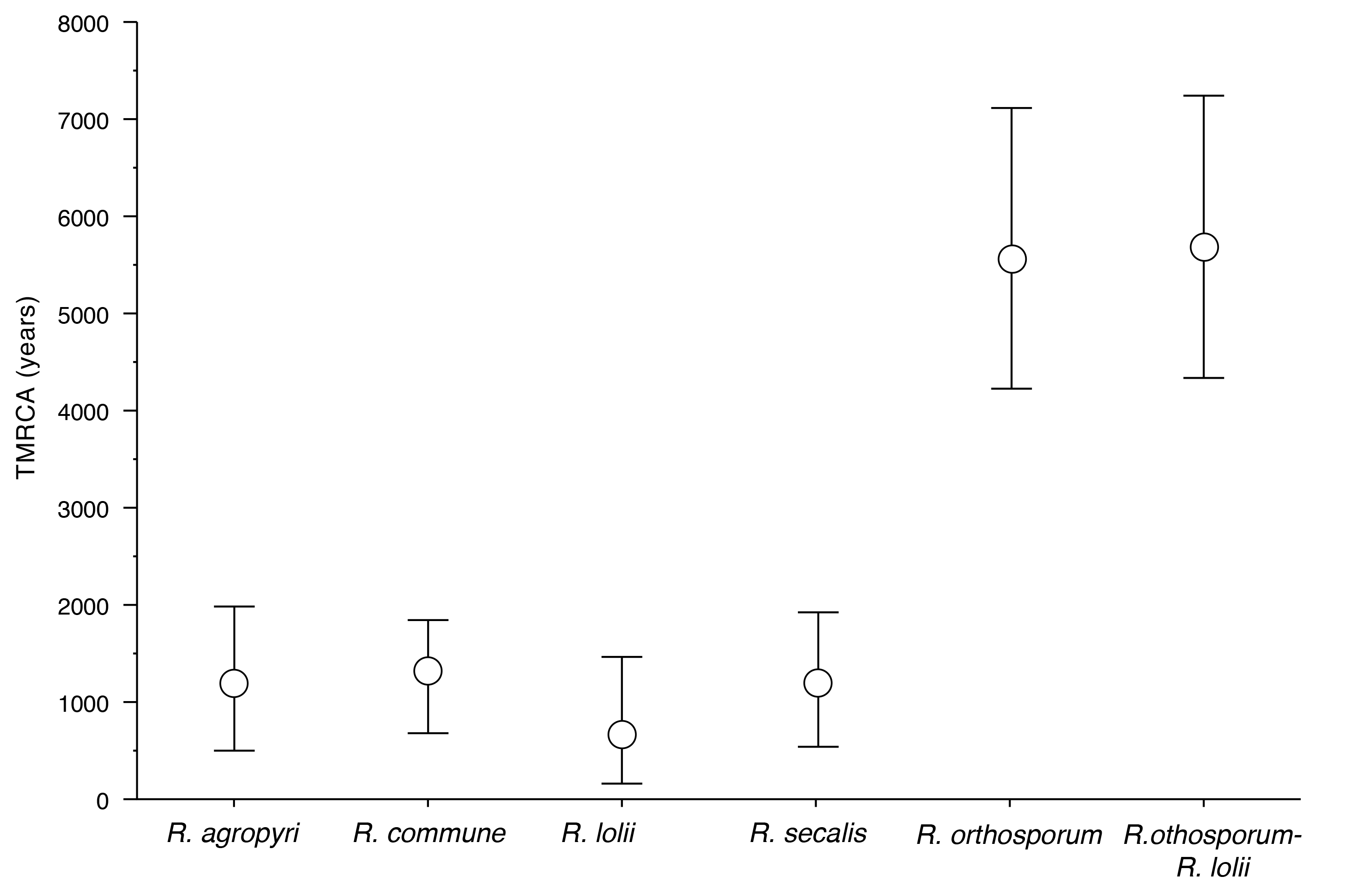

Supplement: Figure S1 — Estimates of time to most recent common ancestor (TMRCA) for the five Rhynchosporium species. Estimates were inferred from the phylogenetic reconstruction shown in Fig. 2. Indicated are mean (○) and 95% credibility intervals (vertical bars, i.e. highest posterior density, HPD). (TIFF) [file pone.0072536.s001.tiff]
