# Supplementary material for: Evolutionary Relationships between Rhynchosporium lolii sp. nov. and Other Rhynchosporium Species on Grasses
Source: PLoS One. 2013 Oct 16;8(10):e72536. doi: 10.1371/journal.pone.0072536 (PMC3797698; doi:10.1371/journal.pone.0072536)
Supplement: Table S1 — Rhynchosporium isolates used for DNA fingerprint testing and spore morphology measurements. (DOCX) [file pone.0072536.s002.docx]

**Table S1**. *Rhynchosporium* isolates used for DNA fingerprint testing and spore morphology measurements.

| Species | Isolate code | Host | Geographical origin | Collected | Spore morphology | RAPD-PCR | rep-PCR |
| --- | --- | --- | --- | --- | --- | --- | --- |
| *R. commune* | 788 | Barley | France | 1997 |  | + |  |
| *R. commune* | K1124 | Barley | UK | Unknown |  | + | + |
| *R. commune* | QUB 12-3 | Barley | Northern Ireland, UK | Unknown |  | + | + |
| *R. commune* | OSA 28-2-2 | Barley | Hertfordshire, UK | 2002 |  | + | + |
| *R. commune* | RS 783 | Barley | UK | 2004 |  | + | + |
| *R. commune* | QUB-30-10 | Barley | Northern Ireland, UK | Unknown |  | + | + |
| *R. commune* | QUB-30-13 | Barley | Northern Ireland, UK | Unknown |  | + | + |
| *R. commune* | R.s. 2310 4.2 | Barley | France | 2008 |  | + |  |
| *R. commune* | R.s. 2313 4.2 | Barley | France | 2008 |  | + | + |
| *R. commune* | R.s. 2314 4.2 | Barley | France | 2008 |  | + | + |
| *R. commune* | R.s. 2318 4.2 | Barley | France | 2008 |  | + | + |
| *R. commune* | GKII 18-3-2 | Barley | Hertfordshire, UK | 2002 |  | + | + |
| *R. commune* | SAC 09/943/14 | Barley | Dundee, UK | 2007 |  | + | + |
| *R. commune* | 19hv09 | Barley | Hertfordshire, UK | 2009 | + | + | + |
| *R. commune* | 53hv09 | Barley | Hertfordshire, UK | 2009 | + | + | + |
| *R. commune* | 62hv09 | Barley | Hertfordshire, UK | 2009 |  | + |  |
| *R. commune* | 73hv09 | Barley | Hertfordshire, UK | 2009 | + | + | + |
| *R. commune* | UK7 | Barley | Aberystwyth, UK | Unknown | + | + | + |
| *R. commune* | D.1.1 | Wall barley | Switzerland | 2004 | + | + | + |
| *R. commune* | E.1.2 | Wall barley | Switzerland | 2004 | + | + | + |
| *R. commune* | 2lm11 | Italian ryegrass | Shropshire, UK | 2011 | + | + | + |
| *R. commune* | 5lm11 | Italian ryegrass | Shropshire, UK | 2011 | + | + | + |
| *R. agropyri* | RS04CG-RAC-A.4.3. | Couch-grass | Switzerland | 2004 |  | + | + |
| *R. agropyri* | RS04CG-RAC-A.5.2. | Couch-grass | Switzerland | 2004 |  | + | + |
| *R. agropyri* | RS04CG-RAC-A.6.1. | Couch-grass | Switzerland | 2004 |  | + | + |
| *R. agropyri* | Rs04CH Rac A.6.1 | Couch-grass | Switzerland | 2004 | + |  |  |
| *R. agropyri* | 1ar10 | Couch-grass | Surrey, UK | 2010 | + | + | + |
| *R. agropyri* | 2ar10 | Couch-grass | Surrey, UK | 2010 |  | + | + |
| *R. agropyri* | 3ar10 | Couch-grass | Surrey, UK | 2010 | + |  |  |
| *R. agropyri* | 4ar10 | Couch-grass | Cluj-Napoca, Romania | 2010 | + |  |  |
| *R. agropyri* | 5ar10 | Couch-grass | Cluj-Napoca, Romania | 2010 | + | + | + |
| *R. agropyri* | 6ar10 | Couch-grass | Cluj-Napoca, Romania | 2010 | + | + | + |
| *R. agropyri* | 7ar10 | Couch-grass | Timisoara, Romania | 2010 |  | + | + |
| *R. agropyri* | 8ar10 | Couch-grass | Nottingham, UK | 2010 | + | + | + |
| *R. agropyri* | 9ar10 | Couch-grass | Nottingham, UK | 2010 |  | + | + |
| *R. agropyri* | 10ar10 | Couch-grass | Nottingham, UK | 2010 | + |  |  |
| *R. agropyri* | 11ar10 | Couch-grass | Nottingham, UK | 2010 | + |  |  |
| *R. secalis* | RS02CH4-2a1 | Rye | Switzerland | 2002 |  | + | + |
| *R. secalis* | RS02CH4-4b1 | Rye | Switzerland | 2002 |  | + | + |
| *R. secalis* | RS02CH4-5a1 | Rye | Switzerland | 2002 |  | + | + |
| *R. secalis* | Rs02CH4-6a.1 | Rye | Switzerland | 2002 | + | + | + |
| *R. secalis* | RS99CH1-H10B | Rye | Switzerland | 1999 |  | + | + |
| *R. secalis* | RS02CH4-13a1 | Rye | Switzerland | 2002 |  | + | + |
| *R. secalis* | RS02CH4-14a1 | Rye | Switzerland | 2002 |  | + |  |
| *R. secalis* | 8.4 | Rye | Russia | 2003 |  | + | + |
| *R. secalis* | 6.2 | Rye | Russia | 2003 | + | + | + |
| *R. secalis* | 4.11.1 | Rye | Russia | 2003 | + | + | + |
| *R. secalis* | 1E7a | Rye | Switzerland | 1999 |  | + | + |
| *R. secalis* | 1B8 | Rye | Switzerland | 1999 |  | + | + |
| *R. secalis* | 1D4a | Rye | Switzerland | 1999 | + | + | + |
| *R. secalis* | I-1a | Triticale | Switzerland | 2002 |  | + | + |
| *R. secalis* | I-2a2 | Triticale | Switzerland | 2002 |  | + | + |
| *R. secalis* | I-3a1 | Triticale | Switzerland | 2002 | + | + | + |
| *R. orthosporum* | 27dg09 | Cocksfoot | Aberystwyth, UK | 2009 | + | + | + |
| *R. orthosporum* | 51dg09 | Cocksfoot | Aberystwyth, UK | 2009 |  | + | + |
| *R. orthosporum* | 52dg09 | Cocksfoot | Aberystwyth, UK | 2009 |  | + | + |
| *R. orthosporum* | 56dg09 | Cocksfoot | Aberystwyth, UK | 2009 | + |  |  |
| *R. orthosporum* | 57dg09 | Cocksfoot | Aberystwyth, UK | 2009 | + |  |  |
| *R. orthosporum* | 58dg09 | Cocksfoot | Aberystwyth, UK | 2009 | + |  |  |
| *R. orthosporum* | 59dg09 | Cocksfoot | Aberystwyth, UK | 2009 | + |  |  |
| *R. orthosporum* | RS04CG-BAR-A.1.1.3 | Cocksfoot | Switzerland | 2004 | + | + | + |
| *R. orthosporum* | RsCH04 Bär A.1.1.3 | Cocksfoot | Switzerland | 2004 | + | + | + |
| *R. orthosporum* | RS04CG-BAR-A.1.1.4 | Cocksfoot | Switzerland | 2004 | + | + | + |
| *R. orthosporum* | RS04ITA D-2.2 | Cocksfoot | Italy | 2004 |  | + | + |
| *R. orthosporum* | RS04ITA D-3.1 | Cocksfoot | Italy | 2004 |  | + | + |
| *R. orthosporum* | RS04ITA D-4.1 | Cocksfoot | Italy | 2004 |  | + | + |
| *R. orthosporum* | RS04ITA D-6.1 | Cocksfoot | Italy | 2004 |  | + | + |
| *R. orthosporum* | RS04ITA D-6.2 | Cocksfoot | Italy | 2004 |  | + | + |
| *R. lolii* | 1lm11 | Italian ryegrass | Shropshire, UK | 2011 |  | + | + |
| *R. lolii* | 3lm11 | Italian ryegrass | Shropshire, UK | 2011 |  | + | + |
| *R. lolii* | 4lm11 | Italian ryegrass | Shropshire, UK | 2011 |  | + | + |
| *R. lolii* | 6lm11 | Italian ryegrass | Aberystwyth, UK | 2011 | + | + | + |
| *R. lolii* | 7lm11 | Italian ryegrass | Aberystwyth, UK | 2011 |  | + | + |
| *R. lolii* | 8lm11 | Italian ryegrass | Aberystwyth, UK | 2011 |  | + | + |
| *R. lolii* | 9lm11 | Italian ryegrass | Aberystwyth, UK | 2011 |  | + |  |
| *R. lolii* | 10lm11 | Italian ryegrass | Aberystwyth, UK | 2011 | + | + | + |
| *R. lolii* | 21lm11 | Italian ryegrass | Shropshire, UK | 2011 | + | + | + |
| *R. lolii* | 22lm11 | Italian ryegrass | Shropshire, UK | 2011 | + | + | + |
| *R. lolii* | 11lp11 | Perennial ryegrass | Aberystwyth, UK | 2011 | + | + |  |
| *R. lolii* | 12lp11 | Perennial ryegrass | Aberystwyth, UK | 2011 | + | + | + |
| *R. lolii* | 13lp11 | Perennial ryegrass | Aberystwyth, UK | 2011 | + | + | + |
| *R. lolii* | 14lp11 | Perennial ryegrass | Aberystwyth, UK | 2011 |  | + | + |
| *R. lolii* | 15lp11 | Perennial ryegrass | Shropshire, UK | 2011 | + | + | + |
| *R. lolii* | 16lp11 | Perennial ryegrass | Surrey, UK | 2011 | + | + |  |
| *R. lolii* | 17lp11 | Perennial ryegrass | Hertfordshire, UK | 2011 |  | + | + |
| *R. lolii* | 18lp11 | Perennial ryegrass | Hertfordshire, UK | 2011 |  | + | + |
| *R. lolii* | 20lp11 | Perennial ryegrass | Hertfordshire, UK | 2011 | + | + |  |
| *R. lolii* | 65lp09 | Perennial ryegrass | Aberystwyth, UK | 2009 |  | + | + |
