# Supplementary material for: Evolutionary Relationships between Rhynchosporium lolii sp. nov. and Other Rhynchosporium Species on Grasses
Source: PLoS One. 2013 Oct 16;8(10):e72536. doi: 10.1371/journal.pone.0072536 (PMC3797698; doi:10.1371/journal.pone.0072536)
Supplement: Table S2 — Primers used for DNA fingerprinting, amplification of gene loci or for discrimination between five Rhynchosporium species. (DOCX) [file pone.0072536.s003.docx]

**Table S2**. Primers used for DNA fingerprinting, amplification of gene loci or for discrimination between five *Rhynchosporium* species.

| Primer name | Purpose | Primer sequence (5'-3') |
| --- | --- | --- |
| DNA fingerprinting ^a^ |  |  |
| OPA-09 | RAPD-PCR fingerprinting | GGGTAACGCC |
| OPAJ-03 |  | AGCACCTCGT |
| OPAJ-06 |  | GCTGGAGTGG |
| OPW-03 |  | GTCCGGAGTG |
| OPW-05 |  | GGCGGATAAG |
| OPW-18 |  | TTCAGGGCAC |
| OPW-19 |  | CAAAGCGCTC |
| ERIC2 ^b^ | rep-PCR genomic fingerprinting | AAGTAAGTGACTGGGTGAGC |
| BOXA1R ^b^ |  | CTACGGCAAGGCGACGCTGAC |
| ERICF |  | ATGTAAGCTCCTGGGGATTCA |
| Amplification of gene loci ^c^ |  |  |
| ATUB-25F | Amplification of alpha-tubulin loci | GAGAAGCTATTAGCATCAACG |
| ATUB-649R |  | CTCCTTTCCAACAGTGTAGTGAC |
| ATUB-540F |  | TCCCTAGAACCATCTACTGCG |
| ATUB-1206R |  | CTTTGGCGGCAGACAACTG |
| ATUB-1103F |  | ACAGTTGTCTCCTCCATTACCG |
| ATUB-1603R |  | TGGACGAAGGCACGCTTAGAG |
| BTUB-21F | Amplification of beta-tubulin loci | ATGCGTGAAATCGTACGTCAC |
| BTUB-615R |  | TGACCGAAAGGACCAGCACG |
| ITS4 | Amplification of ITS loci | TCCTCCGCTTATTGATATGC |
| ITS5 |  | GGAAGTAAAAGTCGTAACAAGG |
| Species-specific diagnostics |  |  |
| LinA-F | *Rhynchosporium commune*-specific | GAGCAATGAACAGTCGGCGCCCCA |
| LinA-R |  | GCTAGGCGGCCTGCCAAAGCAAAG |
| RA6-F | *R. agropyri*-specific | AATCAAAATCTTGATTATAATATACG |
| RA6-R |  | CCAACTTTACTGCCTTGATC |
| RS25-F | *R. secalis*-specific | GGCGGATAAGACGGTAGGTC |
| RS25-R |  | GGCGGATAAGCTTACAGAGA |
| 2RO-F | *R. orthosporum* / *R. lolii* -specific | ACCTCGTTGAAGTAGACG |
| 2RO-R |  | GTGTTCTCTCTAGTGCCG |

^a.^ rep-PCR genomic fingerprinting primers described previously by Versalovic *et al*. [22, 23]; ^b.^ Use of primer pair ERIC2/BOXA1R produces an amplicon of ~400-bp that is specific for *R. lolii* but not *R. orthosporum* isolates; ^c.^ Primer sequences described previously by Zaffarano *et al*. [12].
