# Supplementary material for: Evolutionary Relationships between Rhynchosporium lolii sp. nov. and Other Rhynchosporium Species on Grasses
Source: PLoS One. 2013 Oct 16;8(10):e72536. doi: 10.1371/journal.pone.0072536 (PMC3797698; doi:10.1371/journal.pone.0072536)
Supplement: Table S3 — GenBank accession numbers for sequences obtained in the present study. (DOCX) [file pone.0072536.s004.docx]

**Table S3.** GenBank accession numbers for sequences obtained in the present study.

|  |  |  |  |  | Gene loci |  |  |
| --- | --- | --- | --- | --- | --- | --- | --- |
| *Rhynchosporium* species | **Isolate code** | **Original host** | **Geographic origin** | **Collected** | **alpha-tubulin** | **beta-tubulin** | **ITS** |
| *R. agropyri* | 3ar10 | Couch-grass | Surrey, UK | 2010 | KC819297 | KC819287 | KC819277 |
| *R. agropyri* | 6ar10 | Couch-grass | Cluj-Napoca, Romania | 2010 | KC819298 | KC819288 | KC819278 |
| *R. agropyri* | 10ar10 | Couch-grass | Nottingham, UK | 2010 | KC819299 | KC819289 | KC819279 |
| *R. commune* | 2lm11 | Italian ryegrass | Shropshire, UK | 2011 | KC819300 | KC819290 | KC819280 |
| *R. lolii* | 4lm11 | Italian ryegrass | Shropshire, UK | 2011 | KC819301 | KC819291 | KC819281 |
| *R. lolii* | 7lm11 | Italian ryegrass | Aberystwyth, UK | 2011 | KC819302 | KC819292 | KC819282 |
| *R. lolii* | 13lp11 | Perennial ryegrass | Aberystwyth, UK | 2011 | KC819303 | KC819293 | KC819283 |
| *R. lolii* | 15lp11 | Perennial ryegrass | Shropshire, UK | 2011 | KC819304 | KC819294 | KC819284 |
| *R. orthosporum* | Rs04ITA D-6.2 | Cocksfoot | Italy | 2004 | KC819305 | KC819295 | KC819285 |
| *R. orthosporum* | 59dg09 | Cocksfoot | Aberystwyth, UK | 2009 | KC819306 | KC819296 | KC819286 |
